# Genomic Analysis of Hair Sheep From West/Central Africa Reveals Unique Genetic Diversity and Ancestral Links to Breed Formation in the Caribbean

**DOI:** 10.1111/mec.17796

**Published:** 2025-06-02

**Authors:** Pamela Wiener, Juliane Friedrich, Melissa M. Marr, Gustave Simo, Vincent N. Tanya, Keith T. Ballingall, Pavel Flegontov, Benjamin D. Rosen, Guillaume Sallé, Gordon Spangler, Curtis P. Van Tassell, Mazdak Salavati, Félix Meutchieye, Emily L. Clark

**Affiliations:** ^1^ Roslin Institute and Royal (Dick) School of Veterinary Studies University of Edinburgh Edinburgh UK; ^2^ Department of Biochemistry, The Faculty of Sciences The University of Dschang Dschang Cameroon; ^3^ Cameroon Academy of Sciences Yaoundé Cameroon; ^4^ Moredun Research Institute Midlothian UK; ^5^ Department of Biology and Ecology, Faculty of Science University of Ostrava Ostrava Czech Republic; ^6^ Animal Genomics and Improvement Laboratory Agricultural Research Service, United States Department of Agriculture Beltsville Maryland USA; ^7^ University of Tours, ISP, INRAE Nouzilly France; ^8^ Dairy Research Innovation Centre South and West Faculty, Scotland's Rural College Dumfries UK; ^9^ Department of Zootechnics, Faculty of Agronomy and Agricultural Sciences University of Dschang Dschang Cameroon

**Keywords:** admixture, breed development, hair sheep, local ancestry mapping, population genomics

## Abstract

Cameroon Blackbelly sheep are a domestic breed of hair sheep from West/Central Africa. They are popular with small‐holder farmers in Cameroon as they are highly resilient to local environmental challenges and are prolific a‐seasonal breeders. The aim of this study was to characterise the genetics of Cameroon Blackbelly sheep in relation to global sheep populations and to investigate their relationship to Caribbean hair sheep. We first examined the genetic diversity of the Cameroon Blackbelly breed relative to global sheep populations using 50K SNP data. We also used whole genome sequence data to further investigate relationships between Cameroon Blackbelly and breeds from Africa and Europe, as well as the Barbados Blackbelly breed from the Caribbean, which is phenotypically similar to Cameroon Blackbelly. ADMIXTURE results based on 50K and WGS data demonstrated both West/Central African and European ancestries for the Barbados Blackbelly sheep. Results from *f*_*4*_‐statistics‐based *qpAdm* analyses supported these findings. Local ancestry inference identified several genomic regions in Barbados Blackbelly with high proportions of West/Central African ancestry. One of these, on OAR3, includes various keratin genes, suggesting that these genes may play a role in the shared coat phenotypes of the Barbados Blackbelly and Cameroon Blackbelly. This result is consistent with previous reports of adaptive introgression of coat characteristics in both wild and domesticated species. The findings of our study support the view that sheep were transported from West/Central Africa to the Caribbean as part of the transatlantic slave trade and European colonisation, similar to introductions proposed for cattle and goats.

## 1 Introduction

Over the last few decades, great progress has been made in characterising genetic diversity in the major livestock species (FAO 2015). However, the main focus has been on breeds from temperate regions, with far less focus on livestock from tropical regions. Most of The Republic of Cameroon, located in West/Central Africa, has a tropical climate, with wet and dry seasons. It has a diverse geography, including highlands, low‐lying coastal plains and tropical forests. As such, populations of indigenous livestock have adapted to a range of local environmental conditions. These environmental pressures can shape the genetic diversity of populations of indigenous livestock and give rise to unique genotypic and phenotypic characteristics. Cameroon Blackbelly sheep are popular with smallholder farmers in Cameroon and, with other indigenous small ruminant breeds, make a significant contribution to the national and local economy in Cameroon as a source of meat, milk and fibre (Tendonkeng et al. 2013).

An important recent application of genomic technologies is to analyse genetic diversity patterns to help identify genes and functional gene groups associated with adaptation to local environments. This approach has been applied in various livestock species, for example, reviewed in Woolley et al. (2023) for sheep, and has led to the identification of candidate genes related to adaptation to high altitude (Friedrich and Wiener 2020) as well as other climatic and disease factors, for example, hot or cold temperature (Yurchenko et al. 2018), aridity (Wiener et al. 2021), nematode resistance (Ahbara et al. 2021) and trypanotolerance (Hanotte et al. 2003; Noyes et al. 2011). The Cameroon Blackbelly sheep is a prolific a‐seasonal breeder that is resilient to local health challenges and extremes of environment in Cameroon (Meka et al. 2021). These resilience characteristics are desirable, particularly in the face of changing climates, and may have contributed to the influence of these breeds in shaping breed formation on other continents (Spangler et al. 2017).

**FIGURE 1:**
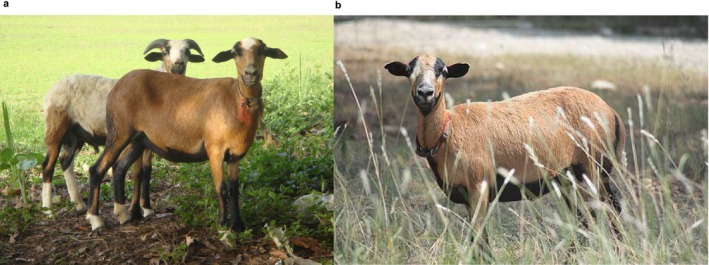
Images of (a) Cameroon Blackbelly and (b) Barbados Blackbelly sheep, illustrating the striking similarity between the two breeds. (a) by Martin Arthur Meka Zibi II. (b) by Bradley Bishop, reproduced under a Creative Commons Attribution‐ShareAlike 4.0 International license (CC BY‐SA 4.0), https://commons.wikimedia.org/w/index.php?curid=34981923.

Based on striking similarities in physical appearance (Figure 1), for example, the black belly and ‘hair’ phenotypes, historical information (Meka Zibi et al. 2019), and genetic findings for other West/Central African sheep (Spangler et al. 2017), it has been suggested that the genetics of the Cameroon Blackbelly sheep have contributed to the formation of the sheep breeds of the Caribbean, such as the Barbados Blackbelly. It is well‐known that European settlers brought European sheep breeds to the Caribbean in the colonial period and it has been proposed that sheep from West/Central Africa were independently transported to this region as part of the transatlantic slave trade, such that some Caribbean breeds have both African and European genetic ancestry (Combs 1983; Spangler et al. 2017). Resolution of these relationships at the individual breed level is more difficult and despite their striking physical similarities (Figure 1), the genetic contribution of the Cameroon Blackbelly to the Barbados Blackbelly has to date been speculative (Meka Zibi et al. 2019).

Understanding the genetic contribution of the Cameroon Blackbelly sheep to the Barbados Blackbelly is important to: (i) evaluate the contribution that the Cameroon Blackbelly sheep as a breed has played to the formation and adaptation of modern sheep breeds in the Caribbean and (ii) assess the impact of West/Central African sheep genetics to other continents via further dissemination of breeds across the globe. Regarding (i), the Barbados Blackbelly has been shown to be comparatively more resistant to primary infection with *Haemonchus contortus* than European production breeds (Aumont et al. 2003; Terefe et al. 2007), and has a wider thermal tolerance range due to its hair coat; these phenotypes may be related to West/Central African ancestry (de Almeida 2018). Regarding (ii), in the United States, hair sheep are a popular component of modern ‘synthetic’ breeds, such as the Katahdin, which have partial Caribbean ancestry and are bred for sustainability traits such as thermal tolerance and resistance to GI helminths (Becker et al. 2020). Synthetic breeds like the Katahdin are likely to become increasingly popular as climate‐related pressures increase on sheep producers (Thorne et al. 2021).

A better understanding of the genetic diversity of indigenous West/Central African breeds, such as Cameroon Blackbelly sheep, will help to facilitate characterisation of the genetic basis of environmental adaptation, disease resistance and sustainability in sheep breeds with West/Central African ancestry. It will also ensure that the unique and important West/Central African sheep breeds, which have contributed to the formation of sheep breeds across the globe, are adequately represented in new and existing genotyping tools and other genomic resources for sheep (Woolley et al. 2023). In this study, we first examined the genetic diversity of the Cameroon Blackbelly sheep relative to global sheep populations using the sheep HapMap dataset (Kijas et al. 2012) and 50K genotypes from Cameroon Blackbelly sheep collected from different geographical locations in Cameroon. Using a combination of data generated for this study and publicly available whole genome sequencing datasets, we then compared the Cameroon Blackbelly sheep to other sheep breeds in Africa, and then to breeds from Europe and the Caribbean, before examining the ancestry of the Barbados Blackbelly. Prior to this study, no genetic studies had been performed to directly identify a unique contribution of the Cameroon Blackbelly sheep to the hair sheep in the Caribbean, and in particular to the Barbados Blackbelly. Our study aims to characterise the genetics of the Cameroon Blackbelly sheep in relation to other sheep breeds from across the globe and to clarify the West/Central African and European ancestry of Caribbean hair sheep, first reported by Spangler et al. (2017).

## 2 Methods

### 2.1 Sample Collection From Cameroon Blackbelly Sheep

**FIGURE 2:**
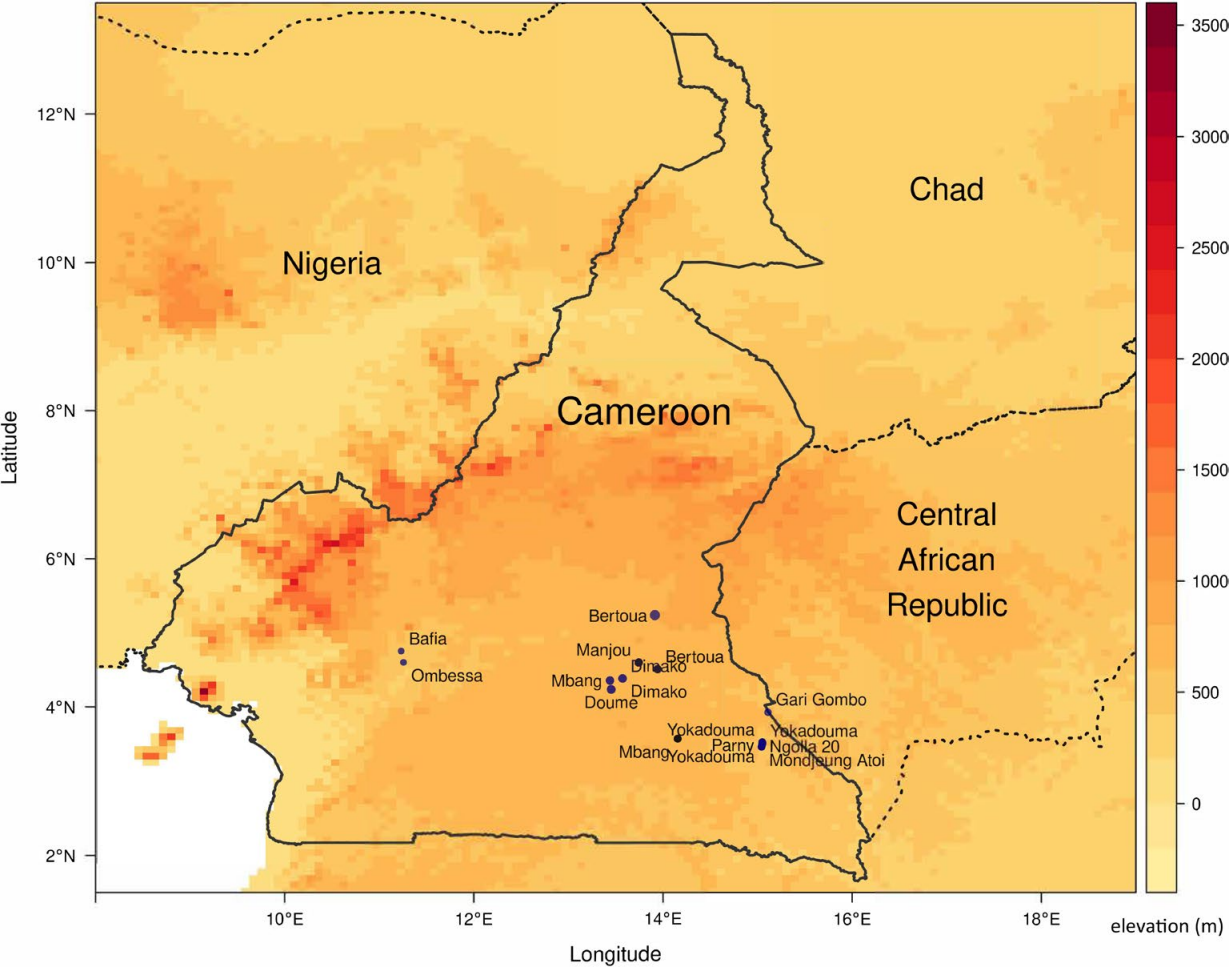
Geographical distribution of farms in Cameroon where the Cameroon sheep genotypes were collected. The name of the village is indicated next to the longitude and latitude of the sampling site. Colour coding refers to altitude.

Ear notch samples were collected from 144 Cameroon sheep from 16 geographic localities in southern and central Cameroon, in collaboration with the University of Dschang (Figure 2). Details of all of the Cameroon Blackbelly samples included in the study, including GPS locations, sex and any other information that could be collected at the time of sampling are included in Table S1. DNA was extracted from the ear notch samples using the DNeasy Blood and Tissue kit (Qiagen), following the manufacturer's instructions, and quality was confirmed by gel electrophoresis in Cameroon. DNA samples were then shipped to the Roslin Institute, University of Edinburgh (Scotland, United Kingdom) where further quality control was performed using Nanodrop Spectrophotometry (Thermo Fisher Scientific). Of the 144 samples that were collected, 108 were of a sufficient quantity and quality, with a 260/280 ratio of > 1.8, to be genotyped on the Illumina OvineSNP50 BeadChip (version 1) (Table 1). To test reproducibility of the genotyping data, three individuals were genotyped in duplicate and then subsequently removed from the analysis after filtering for relatedness (see below) (Table 1). In addition, three Cameroon sheep from a wildlife park in the United Kingdom were genotyped by NeoGen on the Illumina OvineSNP50 BeadChip, with DNA extracted from Performagene Nasal Swabs (DNAGenotek), according to the method described in Kerr et al. (2024).

**TABLE 1:** Origin of samples included in the analysis of 50K SNP array data in this study.

Study	Breed(s)	Country	Number of samples
This Study	Cameroon sheep	Cameroon	111^a^
	Barbados Blackbelly	Guadeloupe	10
	Cameroon sheep	United Kingdom	3
Ahbara et al. (2019)	11 breeds from Ethiopia	Ethiopia	146
	Hammari	Sudan	18
	Kabashi	Sudan	18
	Libyan Barbary	Libya	22
Spangler et al. (2017)	Djallonke	Nigeria	10
	St Croix	St Croix	12
	Bergamasca	Italy	24
Kerr et al. (2024)	Greyfaced Dartmoor	United Kingdom	20
	Norfolk Horn	United Kingdom	33
	Oxford Down	United Kingdom	23
	Ryeland	United Kingdom	8
	Wiltshire Horn	United Kingdom	12
	Texel × Scottish Blackface	United Kingdom	8
Yaro et al. (2019)	Sahelian	Ghana	5
	Djallonke	Ghana	5
Kijas et al. 2012	74 breeds from across the globe	Global	2957

^a^ Including 108 unique individuals and three duplicated samples that were removed from analyses following filtering for relatedness.

### 2.2 Sample Collection From Barbados Blackbelly Sheep

DNA was extracted from blood from 12 Barbados Blackbelly sheep, from the Caribbean archipelago of Guadeloupe, 10 of which were included in this study (Table 1). These samples were collected for a study performed by INRAE investigating resistance to *H. contortus* infection. The quality and quantity of each sample was confirmed using Nanodrop Spectrophotometry (Thermo Fisher Scientific).

### 2.3 SNP Array Genotyping of Samples From Cameroon and Barbados Blackbelly Sheep

All 108 Cameroon Blackbelly were genotyped using the Illumina OvineSNP50 BeadChip (version 1) at the Clinical Research Facility at the University Of Edinburgh (United Kingdom). The Barbados Blackbelly samples from Guadeloupe were genostyped by Neogen (United Kingdom) on the Illumina OvineSNP50 BeadChip (version 3). Genotyping data was analysed and checked for quality in Plink v.1.9 (Chang et al. 2015).

### 2.4 SNP Array Genotypes Included From Publicly Available Datasets

To facilitate comparison of the Cameroon Blackbelly and Barbados Blackbelly with other sheep breeds from across the globe, Illumina OvineSNP50 BeadChip 50K genotyping data for European, Australasian, Caribbean and African sheep breeds were extracted from the public databases (Table 1). In total, 3321 sheep genotypes were collated from these previously published datasets including the worldwide HapMap dataset (Kijas et al. 2012) and additional data for five native breeds from the United Kingdom (Kerr et al. 2024) and breeds from East Africa (Ahbara et al. 2019), West Africa and the Caribbean (Spangler et al. 2017). For two additional breeds from Ghana in West/Central Africa, the Sahelian and Djallonke, publicly available whole‐genome sequence data (Yaro et al. 2019) were down‐sampled to provide 11–22K markers from the Illumina OvineSNP50 BeadChip. Combining the 10 new 50K genotypes for Barbados Blackbelly from Guadeloupe (mentioned above) for this study with 50K genotypes for 24 Barbados Blackbelly (sampled in Barbados) already in the public domain (Kijas et al. 2012) resulted in 34 total Barbados Blackbelly samples.

### 2.5 Merging of SNP Array Genotype Data

All 50K OvineSNP50 BeadChip genotype datasets were matched to the OAR3.1 (GCF_000298735.2) reference genome coordinates and SNP allele composition using PLINK v.1.9. Briefly, the coordinates against the OAR3.1 reference genome for each of the three versions of the SNP 50K OvineSNP50 BeadChip used in the study were obtained from https://webserver.ibba.cnr.it/SNPchimp/. The SNP ids were used for matching the chip coordinates to OAR3.1 compatible coordinates. Then PLINK commands were used to update the binary format files (*‐‐update‐map*, *‐‐update‐chr and ‐update‐pos*). The BCFtools v.1.19 (Li 2011) plug‐in ‘fixref’ was used to check, correct and fix strand orientation against the sheep reference genome version OAR3.1 (GCF_000298735.2). In PLINK, the WGS variant call formats from Yaro et al. (2019) were down‐sampled to the positions (chromosome, position and SNP ids) compatible with the 50K SNP array sets. BCFtools command ‘isec’ was then used to create a list of shared SNPs between all of the datasets, before merging with BCFtools ‘merge’.

### 2.6 Filtering of SNP Array Genotyping Data

Following filtering for genotype coverage > 0.9 and markers on autosomes, the merged dataset included 45,480 markers and 3445 sheep, including the 3321 from public databases and 124 genotyped within this study. Further filtering for minor allele frequency > 0.01 resulted in 45,452 SNPs (‘filtered_dataset’) (Table 2, Table S2). Marker positions were based on the OAR3.1 reference genome assembly coordinates (GCF_000298735.2).

**TABLE 2:** 50K datasets analysed.

	Samples	Populations	Markers	Filtering description
Filtered_dataset	3445	109	45,452	Autosomal, genotype coverage > 0.9, minor allele frequency > 0.01
Filtered_second_degree dataset	2341	109	45,452	Autosomal, genotype coverage > 0.9, minor allele frequency > 0.01 Removal of > second‐degree relatives
Global_second_degree dataset	630	101	11,624	Autosomal, missing genotype rate < 0.35, genotype coverage > 0.9, minor allele frequency > 0.01, between‐marker *r* ^2^ < 0.5 Representative worldwide samples Removal of > second‐degree relatives
Global_second_degree dataset + additional CAMSHP	683	101	11,624	Autosomal, missing genotype rate < 0.35, genotype coverage > 0.9, minor allele frequency > 0.01, between‐marker *r* ^2^ < 0.5 Representative worldwide samples + additional CAMSHP samples (total = 63) Removal of > second‐degree relatives
African_second_degree dataset	181	24	11,624	Autosomal, missing genotype rate < 0.35, genotype coverage > 0.9, minor allele frequency > 0.01, between‐marker *r* ^2^ < 0.5 Representative samples from African populations Removal of > second‐degree relatives
RFMix	*K = 3* (*3 ancestors*) East African 50 West African 51 European/United Kingdom/Pacific 50 Target (BBB) 33 *K = 6* (*2 ancestors*) West African 51 European/United Kingdom/Pacific 50 Target (BBB) 33		45,452 (phased)	Autosomal, genotype coverage > 0.9, minor allele frequency > 0.01

In order to analyse population structure across the maximum number of populations, a subset of markers was extracted. Initially, markers that were not present in the down‐sampled West/Central African breeds were removed and additional filtering was performed on the remaining dataset (missing genotype rate (per individual) < 0.35, genotype coverage (per marker) > 0.9, minor allele frequency > 0.01 and between‐marker *r* ^2^ < 0.5), resulting in a total of 11,624 markers. Individuals were removed from the dataset to assure that no first‐degree relationships were present, using Plink v.2.0 (Purcell and Chang, n.d.‐b; Chang et al. 2015). In addition, in order to balance the dataset across breeds, a subset of individuals was selected for analysis. For all non‐European + Australasian (Australia/New Zealand) breeds, we retained all samples from populations with ≤ 10 samples. For the set of European + Australasian breeds, 250 representative samples were selected using the Corehunter v3.2.1 method (De Beukelaer et al. 2018), which is designed to sample diverse and representative subsets of a larger dataset. The ‘sampleCore’ function was used, with ‘objective’ set to ‘maximize entry‐to‐nearest‐entry Modified Rogers distance’ and ‘mode’ set to ‘fast’. This approach was taken in order to avoid the dataset being dominated by European/Australasian samples, which considerably outnumbered samples from other regions. For other populations with > 10 samples, 10 individuals per population were selected, again using Corehunter, in order to achieve a balanced dataset, leaving 810 individuals. Finally, in order to capture the breadth of worldwide sheep populations but at the same time, produce a manageable dataset for visualisation and analysis, individuals with second‐degree relationships or greater were subsequently removed, leaving 630 individuals across 101 populations (‘global_second_degree_dataset’). A subset of this dataset (‘African_second_degree dataset’) was generated for further analysis, including only the African individuals (181 in total), divided into geographical regions (North, South, East and West/Central). We also analysed an extended version of this dataset (total = 683), including 63 additional Cameroon Blackbelly sheep. Finally, specifically for *f*_4_‐statistics‐based analyses, individuals with second‐degree relationships or greater were also removed from the high‐density filtered_dataset, leaving 2341 samples across 109 populations (filtered_second_degree dataset).

Summary information is shown in Table 2 and Table S2.

### 2.7 Whole Genome Sequencing of Cameroon Blackbelly (CAMSHP), Barbados Blackbelly (BBB) and Djallonke (DJA) Sheep for This Study

Of the above 108 genotyped Cameroon Blackbelly sheep samples, 48 of these with the highest heterozygosity rates, estimated in Plink v.1.9 (Purcell and Chang, n.d.‐a; Chang et al. 2015), were chosen for whole genome sequencing. Only six of the 10 Barbados Blackbelly DNA samples were of sufficient quantity for whole genome sequencing. In addition, DNA samples from 19 Djallonke from West Africa (9 from Ghana, 10 from Nigeria) were provided by collaborators at the USDA for whole genome sequencing, which were collected as described in Spangler et al. (2017).

Quality control of all DNA samples was performed using a Qubit Fluorometer (Thermo Fisher Scientific) and the Tapestation 2200 (Agilent) to ensure DIN values were > 8. For library preparation, 1 mg of gDNA was sheared to fragments of 450 bp mean size that were blunt ended, A‐tailed, size selected and adapters ligated onto fragment ends according to Illumina TruSeq PCR‐free library preparation kit protocol. The Cameroon sheep (CAM) and Djallonke (DJA) libraries were sequenced on the Illumina HiSeq X platform by Edinburgh Genomics (United Kingdom). The Barbados Blackbelly (BBB) samples were sequenced on the Illumina NovaSeq 6000 by NeoGen (United Kingdom). All libraries were sequenced to a mean coverage of 15x with 150 bp paired‐end reads.

### 2.8 Combination of WGS Data Generated for This Study With Publicly Available WGS Data for Sheep Breeds With Shared Ancestry

A total of 99 animals from three ancestries were considered in the analysis of whole genome sequencing data: European (26 samples from breeds of Spanish, French and British origin, publicly available data), Central and West/Central African (48 CAM and 19 DJA, as described above) and Caribbean (6 BBB, from Guadeloupe, as described above). The list of breeds and the number of animals in each breed is shown in Table 3.

**TABLE 3:** The list of sheep breeds selected for the WGS dataset of 99 animals.

Ancestry	Breed	Number of samples	Source
European/Spanish	Castellena	2	PRJEB14684
European/Spanish	Churra	2	PRJEB14684
European/Spanish	Ojalada	2	PRJEB14684
European/French	Milk Lacune	1	PRJEB14684
European/Australian	Merino	8	PRJNA325682
European/USA	Rambouillet	6	PRJNA324837
European/United Kingdom	Cheviot	1	PRJNA160933
European/United Kingdom	Scottish Blackface	1	PRJNA160933
European/United Kingdom	Dollgelau Welsh Mountain	1	PRJNA160933
European/United Kingdom	Welsh Hardy Speckled Face	1	PRJNA160933
European/United Kingdom	Tregaon Welsh Mountain	1	PRJNA160933
West/Central African	Cameroon Blackbelly	48^a^	PRJNA523711
West African	Djallonke	19^a^	PRJNA523711
Caribbean	Barbados Blackbelly	6^a^	PRJNA1013963

^a^ Generated for this study.

### 2.9 Mapping of WGS Data to the ARS‐UI_Ramb_v2.0 Genome and Variant Calling

All raw fastq files were trimmed using Trim‐Galore v0.6.10 (−q 20) and read quality was examined using FastQC. Trimmed, QC‐passed reads were mapped to the latest sheep reference genome (GCF016772045.1 ARS‐UI_Ramb_v2.0 NCBI v106) using BWA v. 0.7.17 (Li and Durbin 2009) with the following flags (bwa mem ‐M ‐R ‘ReadGroup info’). Mapped and sorted BAM files (samtools v1.10 (Danecek et al. 2021)) were then processed by PicardTools v2.17.11 to mark and remove PCR duplicates (MarkDuplicates REMOVE_DUPLICATES = TRUE). The sorted and duplicate free BAM files were used in GATK v.4 (McKenna et al. 2010) for genomic block variant calling (HaplotypeCaller ‐ERC GVCF mode) for each animal. Autosomal chromosome regions (chr1—chr26) were extracted before merging and joint‐calling was performed with CombineVCFs and GenotypeGVCFs, respectively. The following filtering criteria were applied to the SNP subsets of variants (indels were excluded from this analysis). Filtration criteria steps:

1. filter “QD < 2.0” ‐‐filter‐name ‘QD2’

2. filter “QUAL < 30.0” ‐‐filter‐name ‘QUAL30’

3. filter “SOR > 3.0” ‐‐filter‐name ‘SOR3’

4. filter “FS > 60.0” ‐‐filter‐name ‘FS60’

5. filter “MQ < 40.0” ‐‐filter‐name ‘MQ40’

6. filter “MQRankSum < −12.5” ‐‐filter‐name ‘MQRankSum‐12.5’

7. filter “ReadPosRankSum < −8.0” ‐‐filter‐name ‘ReadPosRankSum‐8’

At the final step, the variants with the PASS filter were extracted using BCFtools v.1.16 (bcftools view ‐f PASS) to create the vcf call set for the cohort of 99 animals, including 41,388,271 autosomal SNPs.

Further filtering for allele number (2), minimum genotyping rate (0.90), minimum minor allele frequency (0.05) and minQ (40) resulted in 24,502,830 SNPs. All individuals had a SNP missingness rate < 0.03. Two datasets for further analysis were generated by linkage disequilibrium (LD) pruning, using Plink v.2.0 (Purcell and Chang, n.d.‐b; Chang et al. 2015) with the ‐‐indep‐pairwise flag (‐‐indep‐pairwise 50 10 0.1 and ‐‐indep‐pairwise 50 10 0.8), resulting in 636,983 and 5,758,322 markers for *r* ^2^ < 0.1 and *r* ^2^ < 0.8, respectively.

### 2.10 Population Structure Analysis

For the 50K genotyping data, Plink v.1.9 (Chang et al. 2015) was used to carry out a principal components analysis (PCA) on the global_second_degree dataset. A Neighbour‐Net network for the global_second_degree dataset was constructed in Splitstree v.4.19.2 (Huson 1998) from a genetic distance matrix of pairwise F_ST_ values generated using Plink v.2.0 (‐‐fst CATPHENO method = wc) (Purcell and Chang, n.d.‐b; Weir and Cockerham 1984; Chang et al. 2015). ADMIXTURE software (Alexander et al. 2009) was used to estimate ancestry separately for the global_second_degree dataset (and the version with additional CAMSHP) and for the African_second_degree dataset. Assessment of the number of underlying clusters (K) was assessed using a 15‐fold cross‐validation procedure. The *qpAdm* algorithm from Admixtools 2 (Maier et al. 2023), implemented in R (admixtools 2.0.4, using default parameters), was also used to independently test the plausibility of different simple admixture models and estimate admixture proportions. For a detailed overview of the *qpAdm* method (Haak et al. 2015) and extensive special terminology, see Flegontova et al. (2025) (in particular, Box 1) and Harney et al. (2021). Briefly, the *qpAdm* method is traditionally used in archaeogenetics to test sets of alternative simple (composed of one to four sources) admixture models for a target group of individuals, with the aim of finding a narrow set of optimal models. *QpAdm* tests rely on a matrix of *f*_*4*_‐statistics *f*_*4*_ (*‘left’*_*i*_, *‘left’*_*j*_; *reference*_*i*_, *reference*_*j*_), where the ‘left’ set is composed of the target and sources in a given model, and the (usually larger) reference set is often termed the ‘right’ set. We chose to use *qpAdm* rather than individual *f*_*4*_‐statistics, which often cannot be interpreted unambiguously, especially when they are close to 0, and also do not allow estimation of admixture fractions. Since by default admixture fraction estimates in the *qpAdm* algorithm are not restricted to [0, 1], model plausibility is usually assessed using ‘composite criteria’ relying on both estimated admixture fractions and model *p*‐values, and details of those criteria vary from study to study (Harney et al. 2021; Flegontova et al. 2025). In this study, models in which admixture proportions (weights) ranged between 0 and 1 were considered plausible, following the standard practice in the archaeogenetic literature (Flegontova et al. 2025). We used a *p*‐value threshold (0.01) as only a secondary plausibility criterion as it has been shown that there is poor correlation between *p*‐values and admixture model optimality for both isolation‐by‐distance landscapes and graph‐like genetic histories (Flegontova et al. 2025) and *qpAdm* may be overly conservative in rejecting models (Harney et al. 2021).

For the WGS data, prior to population structure analyses, 22 individuals were identified and removed from the dataset, using Plink v.2.0 (Purcell and Chang, n.d.‐b; Chang et al. 2015), to assure that no second‐degree relationships were present. PCA, Neighbour‐Net and ADMIXTURE analyses were then performed on the remaining 77 samples, using the marker dataset pruned for *r* ^2^ < 0.1, as described above for the 50K analyses.

### 2.11 Local Ancestry Inference of BBB Sheep and Signatures of CAMSHP Ancestry

RFMix v.2 software (Maples et al. 2013) (https://github.com/slowkoni/rfmix) was used to track the ancestry of individual genomic regions in BBB sheep, which had previously been shown to have both European and West African origins (Spangler et al. 2017). Thus, the analyses were conducted to track European and West/Central African genomic segments within BBB genomes. Balanced numbers of individuals were included in the ancestor datasets, as recommended in the documentation for RFMix v.1. For the 50K data, the filtered_dataset (45,452 markers) was phased using Beagle v.5.0 (Browning and Browning 2007; Browning et al. 2018) across the complete set of 3445 individuals (with options impute = false, ne = 1000) as phasing accuracy is known to increase with sample size (Browning et al. 2021). For ancestry inference, BBB (*n* = 33, one was removed due to a high missing genotype rate) were considered as ‘query’ and two different sets of breeds from Europe/Britain and Ireland (hereafter, ‘Britain&Ireland’)/Australasia and West/Central Africa were considered as ancestries based on ADMIXTURE results (see Section 3). The WGS dataset, including all 99 samples and the set of markers (~24 M) not pruned for LD, was phased using Beagle v.5.0, as described above. The marker set pruned for *r* ^2^ < 0.8 was used for RFMix analysis. For ancestry inference, BBB (*n* = 6) were considered as ‘query’ and, as for the 50K data, two different sets of European and West/Central African samples were considered as ancestries based on ADMIXTURE results (see Section 3). For both the 50K and WGS datasets, only individuals without second‐degree relationships (see Population Structure, above) were considered as ancestors but BBB were not filtered for close relationships (in line with the RFMix documentation, which specifies that such filtering is not necessary). For both the 50K and WGS data, RFMix v.2 was run in the PopPhased mode for the different sets of ancestors using default options (window‐size = 5 SNPs, generations = 8).

RFMix reports local ancestry results separately for the two haplotypes at each window (5 SNPs). Extreme ancestry levels were defined as those windows in which a high proportion of the query BBB breed had high levels of ancestry to a specific ancestor at either haplotype. If windows with extreme proportions of a specific ancestry were within 50 kb of each other, they were grouped together into a region. The area ± 50 kb around each identified window or region was scanned for candidate genes using Bedtools (Quinlan and Hall 2010), based on the *Ovis_aries* Oar_v3.1.101 genome annotation for the 50K data and ARS‐UI_Ramb_v2.0 for the WGS data. The OvineSNP50BeadChip was designed using the Oar_v3.1 reference genome build and the sample manifests were based on these coordinates. As such we chose to retain the original assembly positions for the 50K data, instead of lifting them over onto the ARS‐UI_Ramb_v2.0 genome build, to avoid problems of marker instability between reference builds (Ormond et al. 2021). Linkage disequilibrium (*r* ^*2*^) in regions of interest was measured using Plink v.1.9 (Purcell and Chang, n.d.‐a; Chang et al. 2015), with options ‐‐r2, ‐‐ld‐window‐r2 and ‐‐ld‐window‐kb 10.

## 3 Results

### 3.1 Population Structure Analysis of 50K Data

**FIGURE 3:**
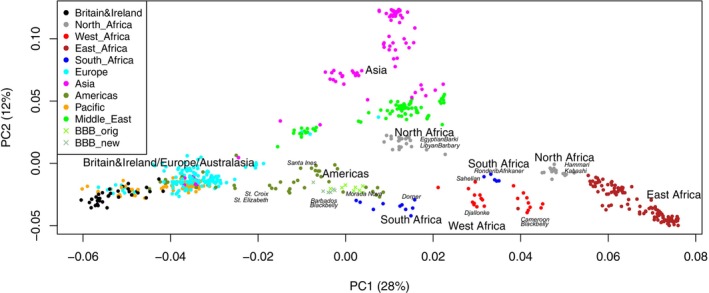
Results of the principal components analysis based on 50K sheep array data for breeds from around the world. The dataset analysed was generated following a stringent quality control procedure, pruning for high linkage disequilibrium, removal of relationships closer than second‐degree and filtering to maintain a representative set of individuals. The final dataset (‘global _second_degree’) included 630 individuals, across 101 populations, and 11,624 SNP markers (see text and Table S2 for further details). Regions and specific populations of interest to the study have been labelled. Of note, several of the populations from the Americas (Barbados Blackbelly, Morada Nova, St. Croix, St. Elizabeth, Santa Ines) cluster in the centre of the plot, between European/Australasian and African populations, while others (Brazilian Creole and Gulf Coast Native, not labelled) are located within the Britain&Ireland/Europe/Australasian cluster.

**FIGURE 4:**
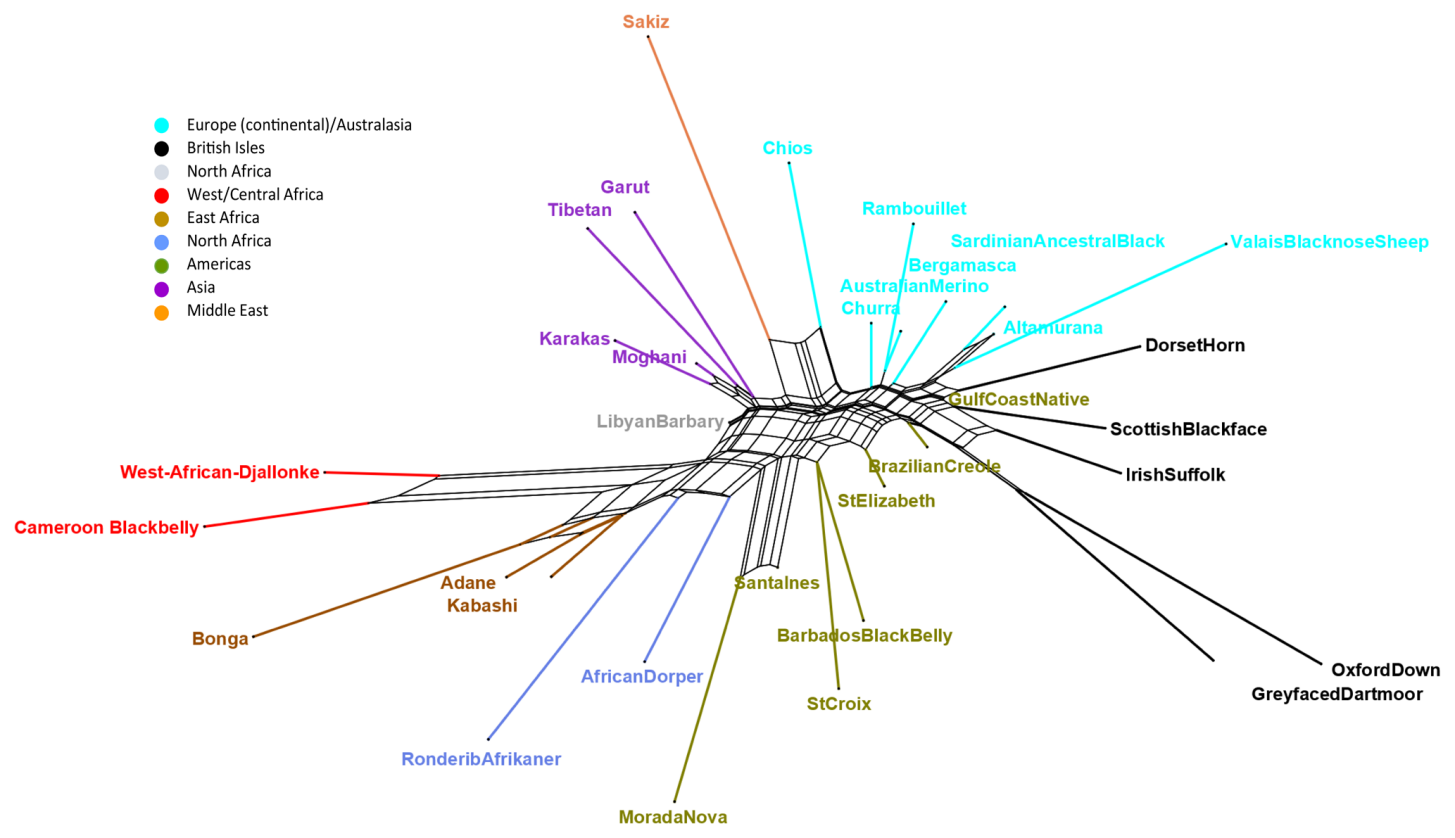
Neighbour‐Net constructed using Splitstree, based on a matrix of pairwise *F*_ST_ values for the global_second_degree dataset (as in Figure 3). A subset of representative breeds was selected to provide a clearer view of relationships.

The first principal component (PC) of the PCA incorporating the 50K data for 101 global sheep breeds (global_second_degree dataset) (Figure 3) explained 28% of the genotype variation while PC2 explained 12%. The separation of individuals/breeds by PCA was strongly associated with geography. Samples of European origin (including those from Europe, Australia and New Zealand) were located at the extreme negative end of PC1 while East African samples were located at the extreme positive end. The samples represented by intermediate PC1 values, with increasing PC1 value, included populations from the Americas, Asia, North Africa, South Africa, West Africa and others from North Africa. PC2 primarily separated Asian, Middle Eastern and some North African populations (high PC2 values) from all other populations (low PC2 values). The Neighbour‐Net analysis (Figure 4, showing a subset of representative breeds to provide a clearer view of relationships) not only supported the geographical clustering seen in the PCA, but also added information on breed relationships that cannot be displayed by a PCA. Reticulation patterns in the network highlighted mixed ancestry in several Caribbean breeds, with evidence of phylogenetic relationships to both West African and European breeds. However, the network showed a large central reticulation between all breeds in the study and, overall, did not fully resolve the complex genetic relationships between sheep breeds in this dataset.

**FIGURE 5:**
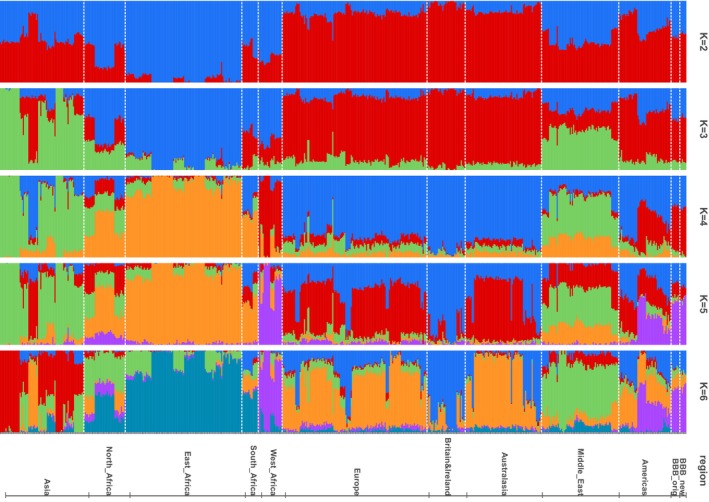
Results of the ADMIXTURE analysis based on the same 50K sheep array data as described in Figure 3. Regarding the region labels, ‘BBB_orig’ refers to Barbados Blackbelly previously sampled in Barbados (Spangler et al. 2017) and ‘BBB_new’ refers to Barbados Blackbelly sampled in Guadeloupe (this study).

The ADMIXTURE analysis of the same dataset as analysed in the PCA showed the lowest cross‐validation error (CVE) for *K* = 14 but began to plateau at *K* = 6 (Figure S1a). The West African sheep formed a distinct cluster starting at *K* = 4 (Figure 5). At *K* = 6, the clusters can broadly be described as West/Central African (purple), East African (teal), Britain&Ireland (blue), other European/Australasian (orange), Middle Eastern (green) and Asian (red), although most regions showed some evidence of admixture. There was strong evidence of both European/Australasian and West/Central African ancestries in several populations from the Americas, including the Barbados Blackbelly (BBB), St. Croix, Morada Nova, Santa Ines and St. Elizabeth breeds. In contrast, the Brazilian Creole and Gulf Coast Native breeds were primarily represented by the two Britain&Ireland/European clusters (blue and orange). These results suggested that the European ancestry in BBB was associated with native British breeds rather than southern European breeds, in that the dominant European cluster found in the BBB was the blue cluster, which is the major cluster in the native British breeds (average assignment probability = 0.87, SE = 0.005) and seen at lower proportion in the other European group (average assignment probability = 0.26, SE = 0.001).

We also performed ADMIXTURE analyses including additional Cameroon (CAM) sheep samples to help choose breeds/samples to include in the subsequent local ancestry analyses. The overall results when additional CAM samples (63 individuals as opposed to 10) were included were broadly similar except that the West/Central African sheep formed a distinct cluster from *K* = 3 rather than *K* = 4 (Figures S1b and S2). The BBB populations showed evidence of admixture for *K* values 2–7. At *K* = 3, BBB populations showed evidence of high ancestry from the red (West/Central Africa) and blue (Europe/Britain&Ireland/Australasian) clusters, with a smaller ancestry proportion from the green (East Africa) cluster. At *K* = 6, BBB populations showed greatest ancestry from the blue (West/Central Africa), green (European) and orange (European) clusters, with a smaller ancestry proportion from the red (Middle East) cluster.

The ADMIXTURE analysis of the African samples (African_second_degree, the African subset of global_second_degree) showed the lowest CVE for *K* = 4 (Figure S3). CAM sheep formed a distinct, well‐defined cluster from *K* = 3 (Figure S4). Higher values of *K* (e.g., *K* = 6) primarily identified further structure in the East African samples.

*QpAdm* was applied to high‐ and low‐density SNP array data to test a variety of two‐ and three‐way admixture models, specifying ‘target’, ‘source’ and ‘reference’ populations (see Flegontova et al. 2025 for an extensive overview of *qpAdm*‐related terminology), where plausibility of each model was assessed as whether admixture proportions (weights) ranged between 0 and 1. In all cases, the target was the merged BBB population. The source populations (putative ancestors of the target) included the West/Central African populations (CAM and/or DJA, separate and merged), Merino populations and UK + Texel populations. The reference populations included a range of combinations of Ethiopian, Middle Eastern, Asian and other European breeds (from 5 to 24 groups in Table S3 and from 4 to 22 groups in Table S4; there were some slight differences in reference sets between the low‐ and high‐density analyses due to filtering‐related differences in breed composition between the global_second_degree and filtered_second_degree datasets). For the higher density dataset filtered to remove close relatives (filtered_dataset_second_degree, 45,452 SNPs, see Table 2), all models in which the merged West/Central African or either CAM or DJA were included as a source population, with either or both Merino and UK + Texel breeds as other source populations, were plausible, except model_4HD (sources = WestAfrica, Merino, UK + Texel; references = Ethiopian, North African, Middle Eastern breeds) (Table S3, where source populations are given in columns C, E, F). Two of these models were also plausible according to a conventional *p*‐value threshold of (>) 0.01, and most models demonstrated low standard errors (1% to 6.6%) of admixture fraction estimates (Table S3), further supporting plausibility of the tested models. Similarly, for the lower density dataset (global_second_degree, 11,624 SNPs), all models in which the merged West/Central African or either CAM or DJA were included as a source population, with either or both Merino and UK + Texel breeds as other source populations, were plausible, except model_19LD (sources = DJA, Merino, UK + Texel; references = Ethiopian and other European breeds) (Table S4, where sources are again given in columns C, E and F). Four models for this dataset were plausible also according to the conventional *p*‐value threshold of 0.01 (Table S4). For both the high‐ and low‐density datasets, none of the models including both CAM and DJA as separate source populations (Table S3: model_20HD—model_25HD, Table S4: model_20LD—model_25LD), with either Merino or UK + Texel as another source population, were plausible.

For many of the admixture models, admixture proportions were greatest for West/Central African breed(s), followed by Merino then UK + Texel, consistent with the ADMIXTURE results. However, other patterns were seen, depending on the choice of reference populations. Taken together, the plausibility and admixture proportion results not only support West/Central African, Merino and UK + Texel ancestries in the target group, but also suggest that the data does not allow us to definitively rank the contributions of the different source populations as differences in *qpAdm* model *p*‐values of few orders of magnitude are often non‐significant (Flegontova et al. 2025).

### 3.2 Population Structure Analysis of WGS Data

PCA was also conducted on the WGS data (636,983 markers) for 77 unrelated European, West/Central African and BBB samples (Table 3; Figure S5) in order to validate patterns seen in the analysis of the 50K data. Based on the previous results of Spangler et al. (2017), publicly available data from breeds of Spanish, French and British origin were used as representatives of the European ancestry. PC1 explained 29% of the variation while PC2 explained 9%. The relationships between the groups were consistent with those seen for the global 50K dataset (Figure 3) such that PC1 discriminated between the three main ancestries and BBB were midway between the European and West/Central African groups. PC2 primarily discriminated between the CAM and DJA populations. PC1 also discriminated between the disparate geographical origins of DJA samples (samples from Nigeria showing PC1 values close to 0.0 and those from Ghana showing PC1 values close to 0.05).

**FIGURE 6:**
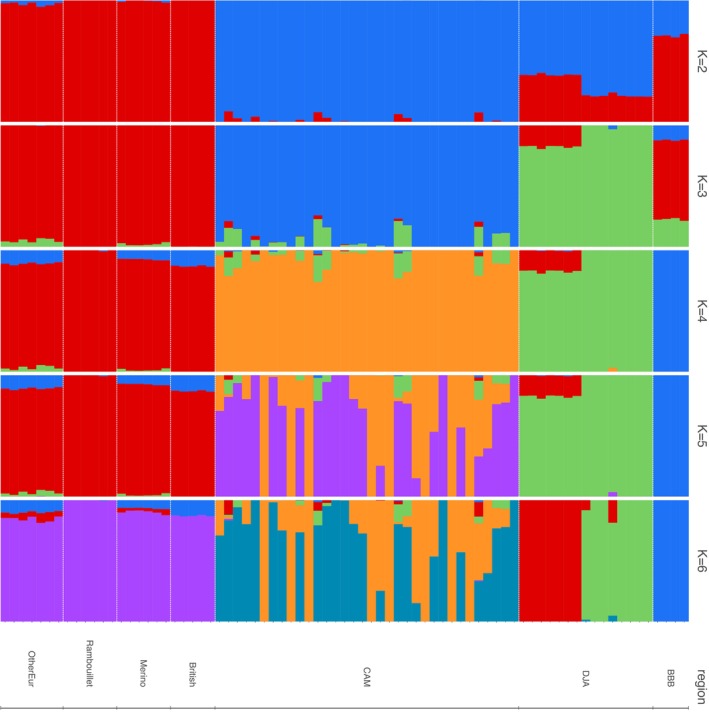
Results of the ADMIXTURE analysis based whole‐genome sequencing data for European, West/Central African and BBB samples. The dataset was generated following a stringent quality control procedure, pruning for high linkage disequilibrium and removal of individuals closer than second degree. The final dataset included 77 individuals and ~637 K SNP markers. Breeds include Cameroon Blackbelly (‘CAM’), Djallonke (‘DJA’), British (Cheviot, Scottish Blackface, Dollgelau Welsh Mountain, Welsh Hardy Speckled Face, Tregaon Welsh Mountain), Merino, Rambouillet and other European (‘OtherEur’: Milk Lacaune, Churra, Ojalada, Castellana).

ADMIXTURE analysis (*K* = 1–7) was conducted using the same WGS dataset as the PCA. There was a fall in CVE after *K* = 1, reaching the lowest point at *K* = 3 and then increasing again from *K* = 4 (Figure S6). At *K* = 3, the defined clusters were associated with European (red), CAM (blue) and DJA (green) samples (Figure 6). In contrast to the 50K results, there was only support for a single European ancestry, while the West/Central African samples encompassed two ancestries (Cameroon, CAM; Djallonke, DJA). The BBB samples showed evidence of ancestry from all three clusters, with European making the greatest contribution. Higher values of K (≥ 4) separated the BBB samples into a separate cluster and subsequently (*K* = 5 and 6) divided the CAM and DJA samples each into two groups. As seen in the PCA, for *K* = 6, the DJA samples were divided by geographical origin (red cluster = samples from Ghana, green cluster = samples from Nigeria). The samples from Ghana also showed evidence of some European ancestry for *K* = 4 and 5; this was not observed in samples from Nigeria. The Neighbour‐Net relationships (Figure S7) also provided evidence of both West/Central African and European ancestry for BBB.

### 3.3 Local Ancestry Inference Based on 50K Data for BBB Sheep and Signatures of West/Central African Ancestry

Two approaches were taken for the RFMix analysis of the 50K data to assess local West/Central African ancestry for BBB and identify genomic regions of high ancestry. The aim was to choose ancestor sets to best represent the potential ancestries for the BBB populations, as observed in the ADMIXTURE results. Thus, we chose ancestor sets based on the results from the analysis of the global_second_degree dataset with additional CAM samples (Figure S2), in order to maximise information for the West/Central African ancestry.

Both approaches were based on the *K* = 6 ADMIXTURE results. In the first approach (two‐ancestry approach), we defined two groups of ancestors for BBB: West/Central African and European/Britain&Ireland/Australasian. For the West/Central African ancestry, we selected individuals (all CAM) with > 0.90 assignment proportion to the blue cluster (total = 51). The selection of the European/Britain&Ireland/Australasian individuals was less straightforward as they showed moderate to high assignment proportions to two clusters (orange and green). A representative sample (total = 50, covering 39 populations; Table S5) of the European/Britain&Ireland/Australasian populations were selected using Corehunter v.3.2.1 (De Beukelaer et al. 2018).

In the second approach (three‐ancestry approach), we defined three groups of ancestors for BBB: West/Central African (blue cluster), European/Britain&Ireland/Australasian cluster 1 (Merino‐related, green cluster) and European/Britain&Ireland/Australasian cluster 2 (British/Texel‐related, orange cluster). For the West/Central African ancestry, using Corehunter (as above), we selected a representative set of 30 CAM individuals from those with > 0.80 assignment proportion to the blue cluster. For the European/Britain&Ireland/Australasian cluster 1 ancestry, using Corehunter (as above), we selected a representative set of 30 individuals with > 0.8 assignment proportion to the green cluster (encompassing five populations, Table S6). For the European/Britain&Ireland/Australasian cluster 2 ancestry, we selected all 30 individuals with > 0.80 assignment proportion to the orange cluster, encompassing eight populations (Table S6).

#### 3.3.1 Two‐Ancestry Approach

**FIGURE 7:**
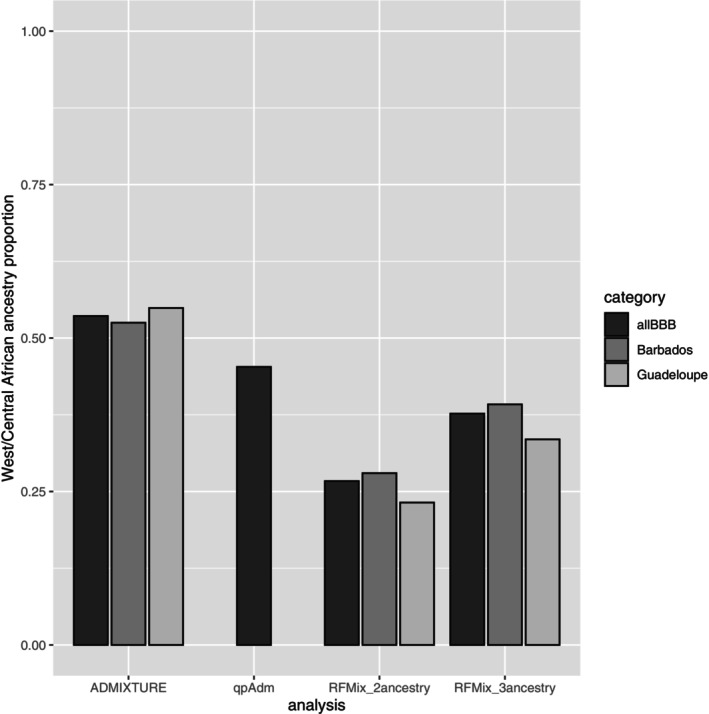
Bar plot showing West/Central African ancestry estimated in several analyses of 50K SNP array data. ADMIXTURE results refer to the average assignment probabilities for Barbados Blackbelly (BBB) sheep to the predominant Cameroon Blackbelly sheep cluster in the *K* = 6 analysis of the global_second_degree dataset (as in Figure 3); *qpAdm* results refer to the average weight estimated for the BBB from several models, based on the (“low‐density”) global_second_degree dataset (Table S4, model1_LD‐model7_LD); RFMix_2‐ and 3‐ancestry results refer to the genome‐wide average maxCAMSHP ancestry for the two‐ and three‐ancestry RFMix models, based on the RFMix dataset (selected set of samples as described in the text, 45,452 SNPs). ‘Barbados’ refers to the BBB samples from the previous study (Kijas et al. 2012), ‘Guadeloupe’ refers to the BBB samples genotyped in the current study and ‘allBBB’ is the merged set of samples.

The genome‐wide averages of CAM (West/Central African) ancestry for the two haplotypes across all BBB individuals were 0.265 and 0.268, with the remaining proportion (0.73) showing Europe/Britain&Ireland/Australasian ancestry. The previously analysed BBB from Barbados (Spangler et al. 2017) had greater overall CAM ancestry (average = 0.280) than those sampled from Guadeloupe in the current study (0.232). For comparison purposes, estimated ancestry proportions are also shown in Figure 7 for this and related analyses.

**FIGURE 8:**
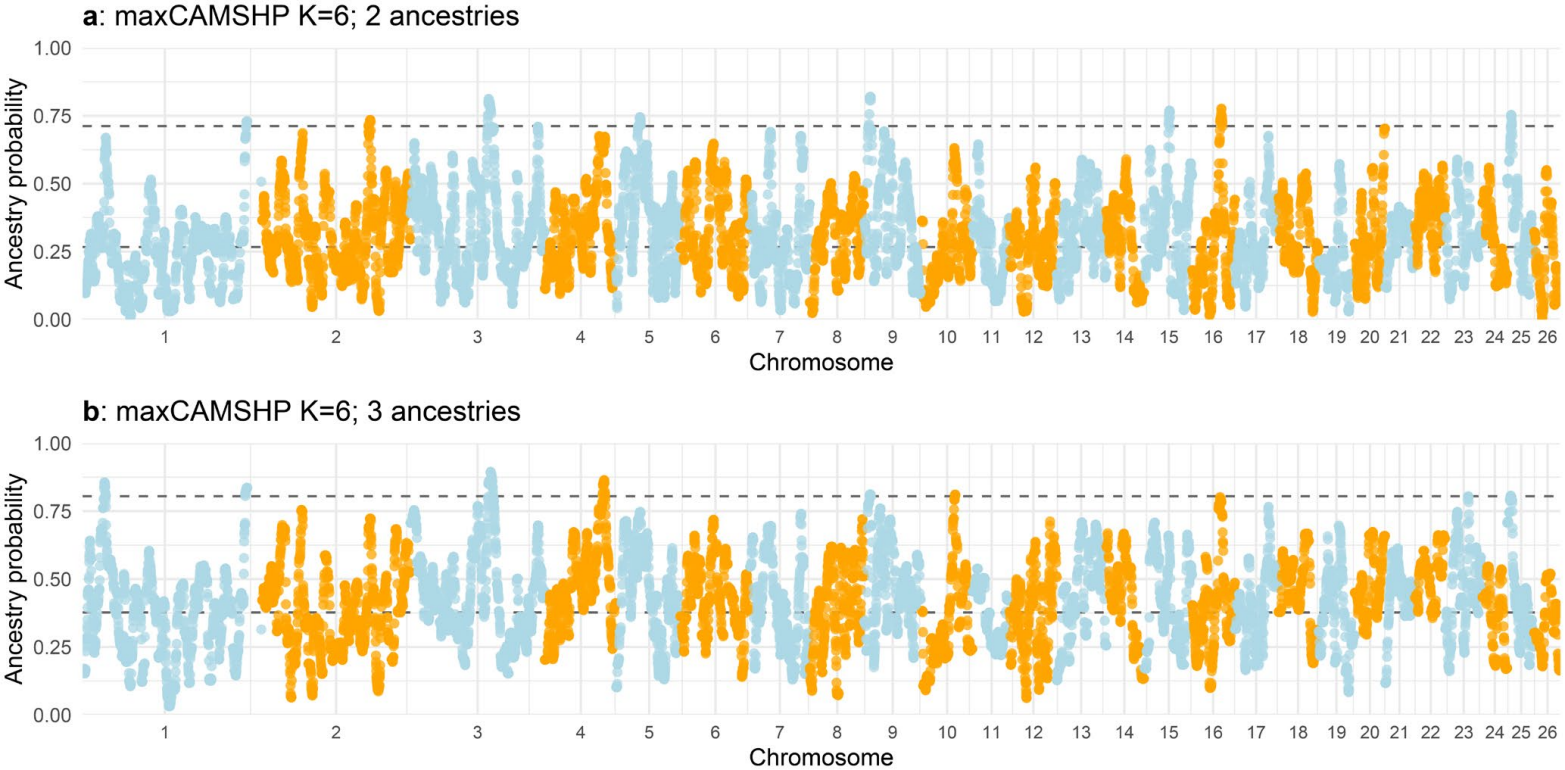
Results of the RFMix analysis based on 50K SNP array data, showing the maximum West/Central African (maxCAMSHP) ancestry across the two haplotypes across the genome. The dataset (‘filtered_dataset’) included 45,452 phased markers. Two‐ and three‐ancestry analyses were performed with a query set of 33 Barbados Blackbelly individuals. (a) Results based on the two‐ancestry analysis: European/Britain&Ireland/Australasian (50 individuals, covering 39 populations, see Table S5 for further details) and West/Central African (51 Cameroon Blackbelly) ancestries. (b) Results based on the three‐ancestry analysis (30 individuals per ancestry): Merino‐related, UK‐Texel‐related and West/Central African (Cameroon Blackbelly) ancestries (see Table S6 for further details).

None of the windows had > 0.90 CAM ancestry for *both* haplotypes in more than 19 of the 33 samples. Furthermore, none of the windows had > 0.90 CAM ancestry for *either* haplotype in more than 31 BBB samples. Windows for which 31 BBB had > 0.90 CAM ancestry for either haplotype were grouped into regions on four chromosomes according to the Oar_v3.1 reference genome (OAR3: 131,815,794–134,161,083, OAR5: 35,604,858–37,373,408, OAR9: 9,669,457–11,252,119 and OAR15: 42,255,552–43,216,426). A total of 117 genes were located within these regions (Table S7), of which 89 were named and unique (Table 4); 58 of these were on OAR3, including several genes from the keratin gene family (REG_4 and REG_5). For visualisation purposes, the maximum CAM (maxCAMSHP) ancestry across the two haplotypes was plotted across the genome (Figure 8a). Regions on OAR3, OAR9 and OAR15 directly overlapped peaks in maxCAMSHP ancestry values and REG_8 on OAR5 was ~85 kb from a peak identified by maxCAMSHP ancestry.

**TABLE 4:** 50K RFMix results: Top CAM ancestry regions for two‐ancestry model.

Region^a^	Chr	Reg_Start^b^	Reg_End^b^	Length	Wind_n^c^	Gene(s)
REG_1	3	131,815,794	132,193,852	378,058	2	*CBX5; COPZ1; GTSF1; HNRNPA1; ITGA5; NCKAP1L; NFE2; PDE1B; PPP1R1A; ZNF385A*
REG_2	3	132,250,928	132,852,500	601,572	3	*ATP5MC2; CALCOCO1; HOXC10; HOXC11; HOXC12; HOXC13; HOXC4; HOXC5; HOXC6; HOXC8; HOXC9; MAP3K12; NPFF; PCBP2; TARBP2*
REG_3	3	132,935,814	133,263,346	327,532	1	*AAAS; AMHR2; EIF4B; ESPL1; IGFBP6; ITGB7; MFSD5; MYG1; PCBP2; PRR13; RARG; SOAT2; SP1; SPRYD3; TNS2; ZNF740*
REG_4	3	133,314,721	133,705,057	390,336	1	*EIF4B; KRT1; KRT3; KRT4; KRT5; KRT71; KRT72; KRT73; KRT74; KRT77; KRT78; KRT79; KRT8*
REG_5	3	133,771,576	134,161,083	389,507	2	*ACVR1B; ATG101; KRT2.11; KRT7; KRT75; KRT80; KRT82; KRT84; KRT85; NR4A1*
REG_6	5	35,604,858	36,361,620	756,762	3	*EIF4E1B; F12; FGFR4; GPRIN1; GRK6; HK3; LMAN2; NSD1; RAB24; RGS14; SLC34A1; SNCB; TSPAN17; UNC5A; ZNF346*
REG_7	5	36,419,513	36,908,510	488,997	2	*B4GALT7; COL23A1; DBN1; DDX41; F12; FAM193B; GRK6; HNRNPAB; N4BP3; NHP2; PDLIM7; PHYKPL; PRR7; RMND5B*
REG_8	5	36,961,824	37,373,408	411,584	2	*CLK4; PROP1; ZNF354A*
REG_9	9	9,669,457	9,780,457	111,000	1	
REG_10	9	9,855,235	10,007,461	152,226	1	
REG_11	9	10,082,948	10,456,093	373,145	2	
REG_12	9	10,666,673	11,252,119	585,446	2	
REG_13	15	42,255,552	42,461,989	206,437	1	*SBF2; SWAP70*
REG_14	15	42,556,060	43,216,426	660,366	3	*AKIP1; ASCL3; C11orf16; DENND5A; IPO7; NRIP3; SCUBE2; SWAP70; TMEM41B; TMEM9B; WEE1; ZNF143*

*Note:* Regions showing > 0.90 CAM ancestry for either haplotype.

^a^ If 5‐SNP windows were within ± 50 kb of each other, they were grouped into a region.

^b^ In bp and defined as window location, according to OAR3.1 (GCF_000298735.2) reference genome.

^c^ Number of 5‐SNP windows in region.

#### 3.3.2 Three‐Ancestry Approach

The genome‐wide averages for the three ancestries across all BBB individuals were the following: CAM (Cameroon Blackbelly) ancestry for the two haplotypes were 0.374 and 0.380, Merino‐related ancestry for the two haplotypes were 0.411 and 0.414 and British/Texel‐related ancestry for the two haplotypes were 0.217 and 0.206. As for the two‐ancestry approach, the BBB from Barbados showed greater CAM ancestry (across haplotype average = 0.392) than BBB sampled from Guadeloupe (average = 0.335) (see Figure 7). Both of the two European ancestries showed lower proportions in the BBB sampled from Barbados (across haplotype, genome‐wide averages: 0.403 for Merino‐related and 0.205 for British/Texel‐related for Barbados BBB vs. 0.438 and 0.228, respectively, for Guadeloupe BBB).

None of the windows had > 0.90 CAM ancestry for *both* haplotypes in more than 24 of the 33 samples. There was a single marker (3:134,008,438 on Oar_v3.1) for which all 33 BBB showed > 0.90 CAM ancestry for *either* haplotype. There were three genes within 50 kb of this position, including KRT7, KRT80 and one unannotated gene (also a keratin‐related orthologue). There were 29 markers for which 32 or 33 BBB showed > 0.9 CAMSHP ancestry for either haplotype. The regions defined by these markers were located on OAR1 (1: 273,457,763–294,082,112), OAR3 (3: 134,008,438–137,101,201) and OAR10 (10: 56,240,111–58,895,153) of the Oar_v3.1 reference genome and 88 genes were found within 50 kb of these regions (Table S8), of which 59 were named and unique (Table 5). The three regions directly overlapped peaks in maxCAMSHP ancestry values across the two haplotypes (Figure 8b). Only the regions on OAR3 overlapped between the two‐ and three‐ancestry models.

**TABLE 5:** 50K RFMix results: Top CAM ancestry regions for three‐ancestry model.

Region^a^	Chr	Reg_Start^b^	Reg_End^b^	Length	Wind_n^c^	Gene(s)
REG_1	1	273,457,763	274,141,473	683,710	3	*SATB1*
REG_2	1	274,195,436	274,648,891	453,455	2	*KCNH8*
REG_3	1	274,740,631	275,128,589	387,958	2	*KCNH8*
REG_4	1	275,183,655	294,082,112	18,898,457	2	*APP2D1*, *EFHB*, *KAT2B*, *RAB5*
REG_5	3	134,008,438	135,084,454	1,076,016	4	*ACVR1B*, *ACVRL1*, *ANKRD33*, *ATG101*, *BIN2*, *CELA1*, *CSRNP2*, *DAZAP2*, *GALNT6*, *KRT7*, *KRT80*, *LETMD1*, *NR4A1*, *POU6F1*, *SCN8A*, *SLC4A8*, *SMAGP*, *TFCP2*, *TMDD1*
REG_6	3	135,322,864	135,498,934	176,070	1	*ATF1*, *DIP2B*, *TMPRSS1*
REG_7	3	135,600,087	135,742,881	142,794	1	*FAM186A*
REG_8	3	135,842,389	136,093,681	251,292	1	*ASIC1*, *COX14*, *FAM186A*, *GPD1*, *LIMA1*, *RACGAP1*, *SMARCD1*
REG_9	3	136,151,883	136,338,867	186,984	1	*AQP2*, *AQP5*, *AQP6*, *FAIM2*, *NCKAP5L*, *RACGAP1*, *TMBIM6*
REG_10	3	136,405,690	137,101,201	695,511	3	*C1QL4*, *CCDC65*, *DNAJC22*, *FAM186B*, *FKBP11*, *FMNL3*, *KCNH3*, *KMT2D*, *LMBR1L*, *MCRS1*, *PRKAG1*, *PRPF40B*, *RHEBL1*, *RND1*, *TMBIM6*, *TROAP*, *TUBA1B*, *TUBA1C*, *WNT1*
REG_11	10	56,240,111	57,197,711	957,600	4	
REG_12	10	57,261,965	57,430,904	168,939	1	
REG_13	10	57,485,800	57,675,612	189,812	1	
REG_14	10	57,735,400	58,895,153	1,159,753	3	

*Note:* Regions showing > 0.90 CAM ancestry for either haplotype.

^a^ If 5‐SNP windows were within ± 50 kb of each other, they were grouped into a region.

^b^ In bp and defined as window location, according to OAR3.1 (GCF_000298735.2) reference genome.

^c^ Number of 5‐SNP windows in region.

### 3.4 Local Ancestry Inference Based on WGS Data for BBB Sheep and Signatures of CAM Ancestry

A follow‐up RFMix analysis was performed using WGS data, but due to limited availability of samples and existing data, this was based on fewer samples and a narrower set of breeds for the European ancestry than that of the 50K data. Again BBB (only six samples in contrast to the 33 in the 50K analysis) were considered as the query set of individuals, with African and European samples as the reference ancestries. As for the 50K analysis, we used the results from the ADMIXTURE analysis to define the ancestor sets to best represent the potential ancestries for the BBB population and the numbers of individuals included as ancestors was balanced within each analysis. Genomic regions of high West/Central African ancestry were based on the ARS‐UI_Ramb_v2.0 assembly.

Again, two approaches were taken to assess local West/Central African ancestry of BBB, based on different models of ancestry. In the first (two‐ancestry approach), two groups of ancestors were defined: West/Central African (CAM/DJA) and European (Spanish/French/British). In order to provide balanced numbers of ancestors, all European samples (24, the smaller set of samples) were included and 24 representative West/Central African samples were chosen using Corehunter (as above) (Table S9). In the second (three‐ancestry) approach, three groups of ancestors were defined: CAM, DJA and European. This differed from the 50K analysis because the ADMIXTURE analysis of the WGS data supported two West/Central African ancestries rather than two European ancestries. In this case, DJA was the smallest set of samples (15) so all of these were included and 15 representative CAM and 15 representative European samples were chosen using Corehunter (as above) (Table S10).

For the two‐ancestry model, the genome‐wide ancestry averages for the two haplotypes across all BBB individuals were 0.26 and 0.27 (average = 0.26) for West/Central African ancestry and 0.41 and 0.40 for European ancestry. For the three‐ancestry model, the genome‐wide ancestry averages for the two haplotypes across all BBB individuals were 0.15 and 0.14 for CAM ancestry, 0.22 and 0.21 for DJA ancestry and 0.63 and 0.64 for European ancestry.

The WGS analyses showed more extreme signatures of ancestry than the 50K analysis. Thus, a more stringent approach was employed to define regions of greatest West/Central African ancestry, based on windows with probability score of 1.00 West/Central ancestry in all six BBBs.

For the two‐ancestry model, 36,868 windows were identified where *both haplotypes* showed probability score of 1.00 West/Central African (AFR = CAM + DJA) ancestry for all six BBBs; these were merged into 52 regions across 20 chromosomes (Table S11) and encompassed 914 genes, 708 of which were named and unique (Table S12). The largest regions were on OAR5 (REG_14 and REG_15, each ~5.1 Mb) and OAR3 (REG_11, ~5 Mb). REG_11 overlapped with that identified in the 50K analyses (both two‐ and three‐ancestry) and REG_15 overlapped with regions identified in the 50K two‐ancestry analysis (REG_6 and REG_7).

For the three‐ancestry model in which CAM and DJA were considered as separate ancestries, 2191 windows were identified where *both haplotypes* showed 1.00 CAM ancestry in all six BBBs. These were merged into two regions on ARS‐UI_Ramb_v2.0: OAR9: 9,130,045–11,339,961, which contained no genes, and OAR19: 31,915,070–34,722,573, which contained 17 genes, encompassing 13 unique gene names (*ARL6IP5*, *EOGT*, *FRMD4B*, *KBTBD8*, *LMOD3*, *MITF*, *ND2*, *rpl23a*, *SUCLG2*, *TAFA1*, *TAFA4*, *TMF1* and *UBA3*). The OAR9 region overlapped regions identified in the 50K analyses but no regions on OAR19 were identified by the 50K analyses. A less stringent criterion was also applied in which 12,150 windows were identified where *either haplotype* showed 1.0 CAM ancestry in all six BBBs. These were merged into nine regions, located on seven chromosomes on ARS‐UI_Ramb_v2.0 (Table 6, Table S13), including regions overlapping the OAR9 and OAR19 regions detected by the more stringent approach. The longest region (REG_4) was on OAR9, but it contained only a single gene (*ADGRB3*). The next longest region (REG_1) was on OAR3 and contained 144 genes, of which 118 were named and unique. This region overlapped those identified by the 50K analyses and included 24 keratin genes, four *HOXC* genes and three *AQP* genes.

**TABLE 6:** WGS RFMix results: Top CAM ancestry regions for three‐ancestry model.

Region^a^	Chr	Reg_Start^b^	Reg_End^b^	Length	Wind_n^c^	Number of genes
REG_1	3	132,564,992	137,567,509	5,002,517	1159	*AAAS; ACVR1B; ADCY6; AMHR2; ANKRD33; AQP2; AQP5; AQP6; ARF3; ASIC1; ATP5MG; BCDIN3D; BIN2; C1QL4; CACNB3; CALCOCO1; CCDC65; CCNT1; CELA1; CSRNP2; DAZAP2; DDN; DDX23; DHH; DIP2B; DNAJC22; EIF4B; ESPL1; FAIM2; FAM186A; FAM186B; FIGNL2; FKBP11; FMNL3; FOXK2; GALNT6; GPD1; HIGD1C; HOXC10; HOXC11; HOXC12; HOXC13; IGFBP6; ITGB7; KANSL2; KCNH3; kmt2d; KRT1; KRT18; KRT2; KRT3; KRT4; krt5; KRT7; KRT71; KRT72; KRT73; KRT74; KRT76; KRT77; KRT78; KRT79; krt8; KRT80; KRT82; KRT83; KRT84; KRT85; Krt86; Krt87; Krt90; LALBA; LARP4; LETMD1; LIMA1; LMBR1L; MAP3K12; MCRS1; METTL7A; MFSD5; MRTFB; MS4A7; NCKAP5L; NPFF; NR4A1; ODF4; pcbp2; PFDN5; POU6F1; PRPF40B; PRPH; PRR13; RAB35; RACGAP1; RARG; RHEBL1; S100A16; SCN8A; SLC11A2; SLC4A8; SMARCD1; SP1; SP7; SPATS2; SPRYD3; TAMALIN; TARBP2; TEX49; TFCP2; TMBIM6; TMPRSS12; TNS2; TROAP; TUBA1A; TUBA1B; WNT1; WNT10B; ZNF740*
REG_2	4	98,723,747	101,236,367	2,512,620	1133	*AGBL3; AKR1B1; BPGM; CALD1; CNOT4; DNAL1; EXOC4; FAM180A; lrguk; MTPN; NUP205; PMP22; SLC13A4; Slc23a4; SLC35B4; STRA8; WDR91*
REG_3	5	52,707,705	56,613,201	3,905,496	1755	*DAD1; DPYSL3; GPR151; GRXCR2; JAKMIP2; KCTD16; LARS1; PLAC8L1; POU4F3; PPP2R2B; PRELID2; rbm27; SH3RF2; STK32A; TCERG1; YIPF5*
REG_4	9	5,949,029	12,554,498	6,605,469	3852	*ADGRB3*
REG_5	9	56,789,680	57,743,068	953,388	360	*FABP5; FABP9; PAG1; PMP2; ZBTB10*
REG_6	9	76,642,941	76,698,346	55,405	37	*SNX31*
REG_7	16	70,858,593	71,890,634	1,032,041	149	*AHRR; BRD9; CCDC127; CEP72; CLPTM1L; EXOC3; IFNLR1; LPCAT1; LRRC14B; NKD2; PDCD6; sdha; SLC12A7; SLC6A18; SLC6A19; SLC6A3; SLC9A3; TERT; TRIP13*
REG_8	19	31,404,702	35,903,145	4,498,443	1433	*ARL6IP5; EOGT; FRMD4B; KBTBD8; LMOD3; LRIG1; MAGI1; MDFIC2; MITF; ND2; rpl23a; SLC25A26; SUCLG2; TAFA1; TAFA4; TMF1; UBA3*
REG_9	20	27,658,834	28,132,079	473,245	1592	*ABHD14B; GABBR1; HNRNPK; MOG; OR2H1; OR2I1P; OR4E2; POLR1H; PPP1R11; RNF39; THG1L; TRIM10; TRIM15; TRIM26; TRIM31; TRIM40; UBD; ZFP57*

*Note:* Regions showing > 1.00 CAM ancestry for either haplotype.

^a^ If 5‐SNP windows were within ± 50 kb of each other, they were grouped into a region.

^b^ In bp and defined as window location, according to ARS‐UI_Ramb_v2.0 NCBI v106 (GCF016772045.1) reference genome.

^c^ Number of 5‐SNP windows in region.

### 3.5 LD Structure of OAR3 Extreme Ancestry Region

We compared *r* ^*2*^ within the extreme West/Central African ancestry region on OAR3, identified from the WGS RFMix analysis, and across the remainder of OAR3; for between‐marker distances less than ~4kbp, *r* ^*2*^ was significantly greater in the extreme West/Central African ancestry region (Figure S8). The high variance in *r* ^*2*^ beyond marker distances of 4 kbp for the extreme ancestry region was due to the small number of marker pairs located in these distance bins.

## 4 Discussion

### 4.1 West African Sheep Breeds Represent Unique Genetic Diversity

This study documented the unique genetic diversity of West/Central African sheep. In the ADMIXTURE analysis of 50K data from global sheep populations, samples from West Africa can be identified as an independent and well‐defined cluster under models of four or more clusters (*K* values). Other major clusters were associated most strongly with East Africa, Europe and Asia. In the ADMIXTURE analysis of only African sheep, a West/Central African cluster was also clearly separated from other samples, demonstrating the genetic distinctiveness of sheep from this region. The Principal Components Analysis (PCA) of the global dataset further demonstrated the unique diversity in the West/Central African sheep, relative to the other breeds analysed and showed genetic differentiation between the Cameroon Blackbelly sheep from West/Central Africa and other West African sheep (‘Sahelian’ and ‘Djallonke’). This was supported by the PCA based on WGS data in which PC2 clearly separated the Djallonke and Cameroon populations.

Our 50K ADMIXTURE results for the Barbados Blackbelly (BBB) sheep from the Caribbean validated those of Spangler et al. (2017), demonstrating both West/Central African and European ancestries. The BBB samples from Guadeloupe, genotyped in the current study, clustered with those BBB presented in Spangler et al. (2017) but showed lower levels of West/Central African ancestry. The Neighbour‐Net analysis also supported mixed ancestry for BBB. Our 50K ADMIXTURE analysis suggested that the European ancestry was more associated with Britain&Ireland breeds than other European (e.g., Spanish) breeds. However, this was not supported by the RFMix results based on the 50K data, in which the average genome‐wide Merino ancestry (0.41) was greater than that of the Britain&Ireland breeds (0.21). The *qpAdm* analysis also supported West African, Merino and Britain&Ireland ancestries but gave varying results for admixture proportions of the European populations, depending on the choice of reference populations, suggesting that our data did not allow us to definitively rank the contributions of the three ancestries. The WGS ADMIXTURE and Neighbour‐Net analyses supported West/Central African and European ancestries in the BBB samples, but did not distinguish between different European (e.g., Merino and Britain&Ireland) ancestries. Further exploration of specific hypotheses (in particular, once additional WGS data is available) may help to clarify the relative contributions of the different ancestries to BBB and other breeds of the Caribbean and the Americas. The finding of West/Central African ancestry in the BBB samples from our study and Spangler et al. (2017) supports the view that sheep were transported from West/Central Africa to the Caribbean as part of the slave trade and European colonisation during the 17th century (Meka Zibi et al. 2019). A similar phenomenon has also been suggested for Creole cattle and goat breeds of the Americas and the Caribbean, which show evidence of both Iberian and African ancestries (Gautier and Naves 2011; Sevane et al. 2018; Ginja et al. 2019; Ben‐Jemaa et al. 2023; Ward et al. 2024). In the case of sheep, it has been hypothesised (Combs 1983) that some physical characteristics of African breeds were better suited to the environmental conditions of the Caribbean than those of European sheep, leading to the development of new breeds with West/Central African features, for example, hair rather than wool phenotype. In the case of Creole cattle from Guadeloupe, the regions showing strong evidence of selection include genes related to immunity, thermotolerance and physical activity, suggesting possible adaptation to tropical environments (Ben‐Jemaa et al. 2023). A potential next step would be to perform a comparative analysis across admixed sheep, cattle and goat populations from the Caribbean and the Americas to test for shared signatures of selection.

### 4.2 Genomic Regions Showing High West/Central African Ancestry in Barbados Blackbelly Sheep

There was some consistency between the genomic regions showing extreme levels of West/Central Africa from the various RFMix analyses performed in BBB sheep, including two different ancestry models each for the 50K and WGS datasets. The most notable region was that on OAR3, which was identified in all analyses. This extended region encompasses many genes, including a number of keratin genes (24 in the three‐ancestry WGS analysis). Keratin and keratin‐associated proteins have been linked to various hair/fleece characteristics as well as coat colour in sheep, goats and other mammals (Kalds et al. 2022). For example, Fan et al. (2013) showed that the top 30 genes that were highly upregulated in skin samples from black and white Sunite sheep included genes of the keratin family. Kang et al. (2013) also identified highly up‐regulated keratin genes in the skin of small tail sheep with curly fleece. The OAR3 region also includes several *HOXC* genes, which are one of the *HOX* gene clusters, key genes that specify regions of the body plan of the animal embryo. Other *HOX* genes have been shown to be associated with various sheep phenotypes, including tail and horn morphology (Kalds et al. 2022).

Other regions of note include those on OAR5, 9 and 19. The extended region on OAR9 was identified as having extreme West/Central African or CAM ancestry in three of the four analyses. However, very few genes are mapped to this region. The region identified in the 50K two‐ancestry and the WGS two‐ancestry analyses did not contain any genes while the region identified in the WGS three‐ancestry analysis only contained a single gene, *ADGRB3* (adhesion G protein‐coupled receptor B3). *ADGRB3* is expressed at high levels in the brain and adrenal gland (Clark et al. 2017) and variants within *ADGRB3* have been associated with divergent faecal egg counts in Katahdin sheep (Becker et al. 2022). The region on OAR19 was identified in both WGS analyses, and (with the OAR9 region) showed the most extreme signature of CAM ancestry in the three‐ancestry model but was not identified in the 50K analyses. The region identified by the three‐ancestry WGS model contains 13 named genes (17 for the two‐ancestry model), including *MITF* (microphthalmia‐associated transcription factor), which has been associated with various pigmentation phenotypes, including white spotting in dogs, cattle and horses (Rothschild et al. 2006; Hauswirth et al. 2012; Hofstetter et al. 2019). In a study of Tibetan sheep, Han et al. (2015) showed that *MITF* is more highly expressed in black coat skin tissue than white. A region on OAR5 was identified in both the 50K and WGS two‐ancestry models. There were 19 named genes in that region that overlapped between the results for the 50K and WGS two‐ancestry models, which include *FGFR4*, a fibroblast growth factor gene which plays an important role in embryonic development, tissue repair, tumour angiogenesis and tumour progression, and *B4GALT7*, in which mutations have been associated with dwarfism in horses (Leegwater et al. 2016).

Our results support the view that the hair and/or coat colour phenotypes of BBB derive from its West/Central African ancestry (Meka Zibi et al. 2019), at least partially accounting for the similarity in appearance of the two breeds. However, further analyses are required to confirm this and to identify which genes control these traits. For example, studies in other admixed Caribbean breeds could help to narrow down the genomic regions associated with specific traits. In addition, gene expression data from different tissues (e.g., hair and skin), comparing BBB, West/Central African hair sheep and wool sheep, would provide key functional information to identify candidate genes driving these characteristics. Evidence of introgression of genes related to coat‐related traits in this study is in line with other studies of introgression in both wild and domesticated species. There are examples in various taxa of adaptive introgression of genomic segments carrying genes associated with coat/skin colour and hair/fur traits (Hedrick 2013), for example, in wolves (Anderson et al. 2009), pigs (Wilkinson et al. 2013), lizards (Feiner et al. 2024) and humans (Vernot and Akey 2014), among many others. There is also clear evidence that these traits have been under strong selection in both wild and domesticated species (Wiener and Wilkinson 2011; Orteu and Jiggins 2020).

Overall, when comparing the 50K and WGS analyses, there were more extreme signatures of West/Central African ancestry in BBB's based on the WGS analyses, in that many regions showed > 1.00 West/Central African ancestry in all BBB's for both haplotypes, whereas none were found in the 50K analyses. This result was not associated with the smaller number of BBB's in the WGS analysis (six samples) as we looked at ancestry based on 50K data for these same six samples and found the same results as for the full sample (33 BBB's). As discussed above, there were notable overlaps in the regions identified as having extreme West/Central African ancestry between the 50K and WGS analyses, in particular, on OAR3 and OAR9. However, the OAR19 region with the most extreme West/Central African ancestry in the WGS analysis was not seen in the 50K analysis, possibly due to reduced marker density in that region. A somewhat surprising finding was that for both OAR3 and OAR9, the regions identified by the WGS analysis were substantially longer than that from the 50K analyses, although the WGS analysis employed a more stringent criterion for identification. It is not clear whether this is related to the greater marker density of the WGS data or the different samples included in the two analyses. As a result of the larger region identified using WGS, the number of candidate genes in the OAR3 region was much greater such that this analysis did not enable us to narrow down the candidates that might be associated with the hair and/or pigmentation phenotypes. Some of the differences between the RFMix results for the 50K and WGS analyses could also be due to the different genome assemblies used to define the marker positions for the two datasets (50K: Oar_v3.1 and WGS: ARS‐UI_Ramb_v2.0). We chose not to lift the coordinates for the 50K analysis onto ARS‐UI_Ramb_v2.0 as this is known to cause misleading results (Ormond et al. 2021). Finally, the substantial differences in data size and composition between the 50K and WGS datasets may contribute to differences in both the population structure and local ancestry results. As WGS datasets accumulate in the future, to better reflect the diversity of sheep worldwide, more robust analyses will be possible.

### 4.3 Sensitivity of Results to Dataset Composition and Assumptions About Ancestors

Results from the local ancestry analyses we performed using RFMix differed based on the assumptions about ancestry. Estimates of West/Central African ancestry were lower for the two‐ancestry than three‐ancestry models in both the 50K (0.27 vs. 0.38) and WGS (0.26 vs. 0.36, sum of CAM + DJA for the three‐ancestry model) analyses. Furthermore, although some regions of extreme West/Central African ancestry overlapped between the two‐ and three‐ancestry analyses, others were not shared. As the three‐ancestry model for the WGS data separated Cameroon Blackbelly and Djallonke ancestries, we view this to be potentially more powerful than the two‐ancestry model for identification of Cameroon Blackbelly origins in the Barbados Blackbelly genome. In contrast to RFMix, the *qpAdm* analyses gave more similar estimates of West/Central African ancestry for models with two source populations (~0.42) and those with three source populations (~0.45).

Other studies have also reported differences in local ancestry results based on model assumptions. In a study on humans (the Brazilian population and other populations from the Americas), Secolin et al. (2019) observed differences in local ancestry results depending on assumptions about reference populations. Under one formulation of a ‘Native American’ parental reference, they found a region on chromosome 8p23.1 with excess Native American ancestry compared to the rest of the genome. However, when they defined the Native American reference in a different way, there was no such signal. In another study of admixture in Brazilians, Escher et al. (2022) also found differences in the inference of the Native American ancestral component of current Brazilian individuals between models assuming three (African, European, Native American) or four (African, European, Native American, East Asian) parental reference populations.

### 4.4 Implications for Breeding in the Face of Changing Climates and Other Pressures

Results from this study may have implications for breed development and improvement in the face of climate change and other future challenges facing livestock production. Selective breeding in order to produce animals with sustainable traits, such as hair instead of fleece in sheep and the slick hair coat trait in cattle (Huson et al. 2014), will lead to extended regions of linkage disequilibrium in the genome, such that alleles from many genes will hitchhike along with the allele(s) directly influencing the traits of interest. Once the genes associated with the trait of interest are identified, it is important to consider what other genes are located nearby and whether these might cause unfavourable selection outcomes due to statistical associations within genomic regions.

Hair sheep are known to be more resistant to GI helminths relative to wool breeds, although the exact genetic mechanism for this remains unknown (MacKinnon et al. 2010); they are also generally highly resilient, thriving in areas of the globe with high levels of pathogen challenge. Future livestock breeding programmes, in both tropical and temperate regions, will need to involve concomitant improvement of multiple sustainability and health traits. For example, introducing sustainability traits, such as wool shedding in sheep, and resilience traits, such as resistance to GI helminths, into modern production breeds will be necessary if predicted climatic changes become reality. New genomic resources such as those generated for this study and studies of other livestock species can help us to understand more about the breed formation of the past (e.g., Da Silva et al. 2025). They can also improve our knowledge of the potential benefits of including highly resilient breeds, such as the Cameroon and Barbados Blackbelly sheep and Creole cattle and goats, in the breeding programmes of the future, and highlight the importance of conserving genetic diversity in livestock.

## Author Contributions

Funding for the project from the GCRF was awarded to Emily L. Clark. Emily L. Clark, Pamela Wiener and Félix Meutchieye conceived the study. Emily L. Clark and Pamela Wiener designed and managed the study. Pamela Wiener, Mazdak Salavati, Juliane Friedrich and Melissa M. Marr performed the data analysis. Pavel Flegontov contributed to the *qpAdm* analysis. Félix Meutchieye and Vincent Tanya coordinated and performed the sample collections for the Cameroon sheep and liaised with the Ministry for Agriculture in Cameroon. Gustave Simo performed the DNA extractions in Cameroon. Keith T. Ballingall, Benjamin D. Rosen, Guillaume Sallé, Gordon Spangler and Curtis P. Van Tassell provided other samples. Pamela Wiener and Emily L. Clark drafted the manuscript with significant input from Juliane Friedrich, Melissa M. Marr and Mazdak Salavati. All authors approved the final version.

## Disclosure

Benefits Generated: A research collaboration was developed with scientists from the United Kingdom, Cameroon, France, and the USA. All collaborators are included as co‐authors. The results of the research have been shared with all collaborators and the broader scientific community (see Data Accessibility Statement). More broadly, our research groups are committed to international scientific partnerships and capacity building. Details of specific collaboration agreements and permissions are included in the Ethics Statement.

## Ethics Statement

Collection of samples for this study, from Cameroon Blackbelly sheep from different geographical localities in Cameroon for genomic analysis, was approved by the Roslin Institute, University of Edinburgh Animal Work Abroad Ethical Review Committee (RI‐AWA‐14). Sample collection was performed under veterinary supervision and the study as a whole was compliant with the requirements of the Nagoya protocol. The work was performed under collaboration agreement (ROSLIN1929) between the University of Edinburgh and the University of Dschang and the samples were exported according to export permit 00696 from MINEPIA, authorising exportation of products of animal origin from Cameroon. The Djallonke samples were collected according to the approvals described in Spangler et al. (2017) and transferred to the University of Edinburgh under material transfer agreement (ROSLIN2033) with the USDA‐ARS. The Barbados Blackbelly samples from Guadeloupe were collected by INRAE, under the supervision of the local INRAE ethics committee.

## Conflicts of Interest

The authors declare no conflicts of interest.

## Supporting information


**Figures** **S1–S8.**

**Tables** **S1–S14.**

## Data Availability

The whole genome sequencing generated for this project for the Cameroon sheep and Djallonke has been deposited in NCBI under Bio Project PRJNA523711 and for the Barbados Blackbelly under PRJNA1013963. The details of the individual accessions for the whole genome sequencing data deposited in the Short Read Archive and analysed in this study are included in Table S14. The Illumina 50K genotypes for the Cameroon Blackbelly and Barbados Blackbelly sheep are deposited in Dryad (https://doi.org/10.5061/dryad.2ngf1vj1k).
