# Supplementary material for: Genomic Analysis of Hair Sheep From West/Central Africa Reveals Unique Genetic Diversity and Ancestral Links to Breed Formation in the Caribbean
Source: Mol Ecol. 2025 Jun 2;34(24):e17796. doi: 10.1111/mec.17796 (PMC12717993; doi:10.1111/mec.17796)
Supplement: Supplementary file 1 — Figures S1–S8. [file MEC-34-e17796-s001.pptx]

## Slide 1
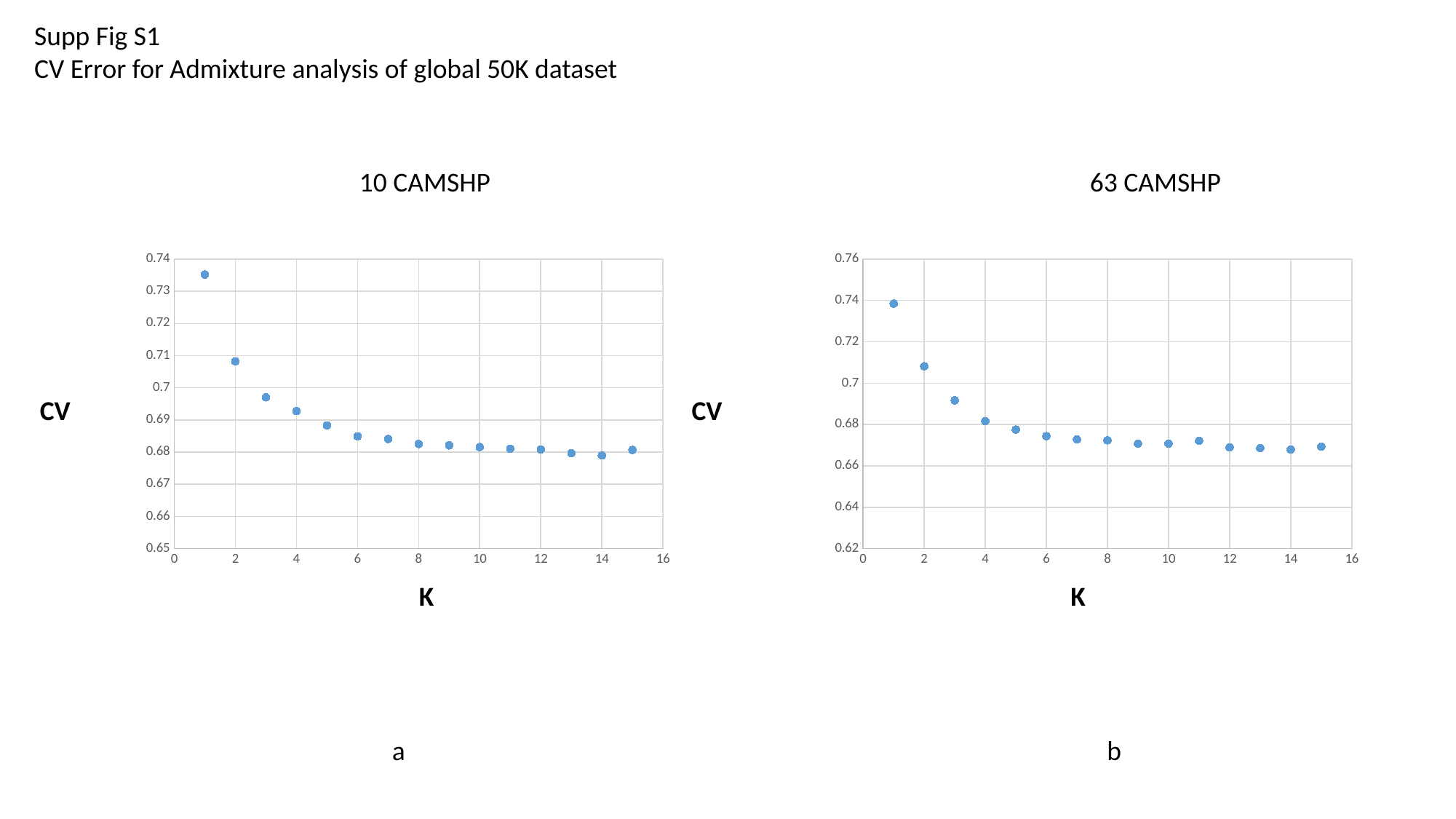

Supp Fig S1
CV Error for Admixture analysis of global 50K dataset
10 CAMSHP
63 CAMSHP
### Chart
| Category | |
|---|---|
### Chart
| Category | |
|---|---|CV
CV
K
K
a
b

## Slide 2
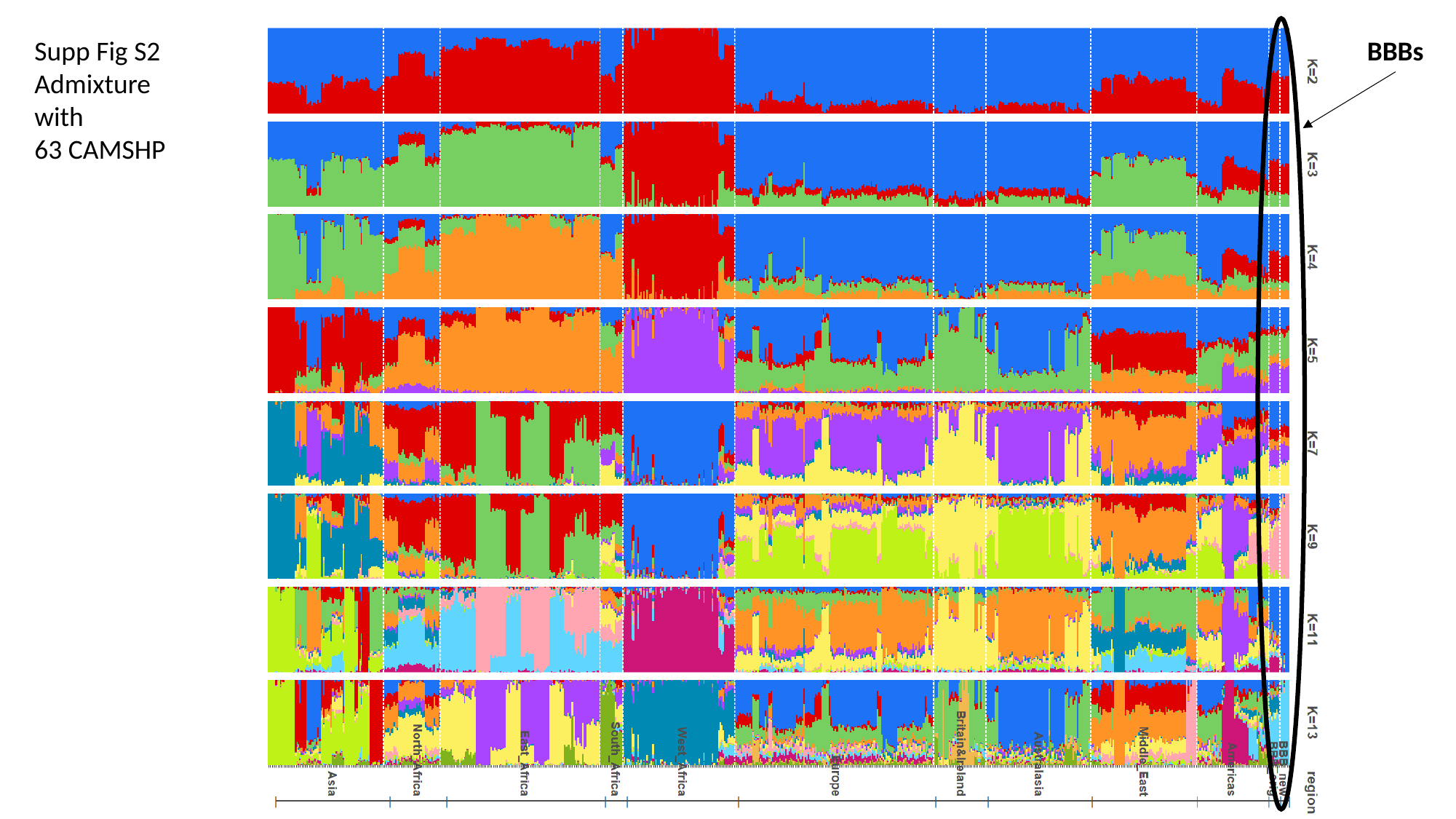

BBBs
Supp Fig S2
Admixture
with
63 CAMSHP

## Slide 3
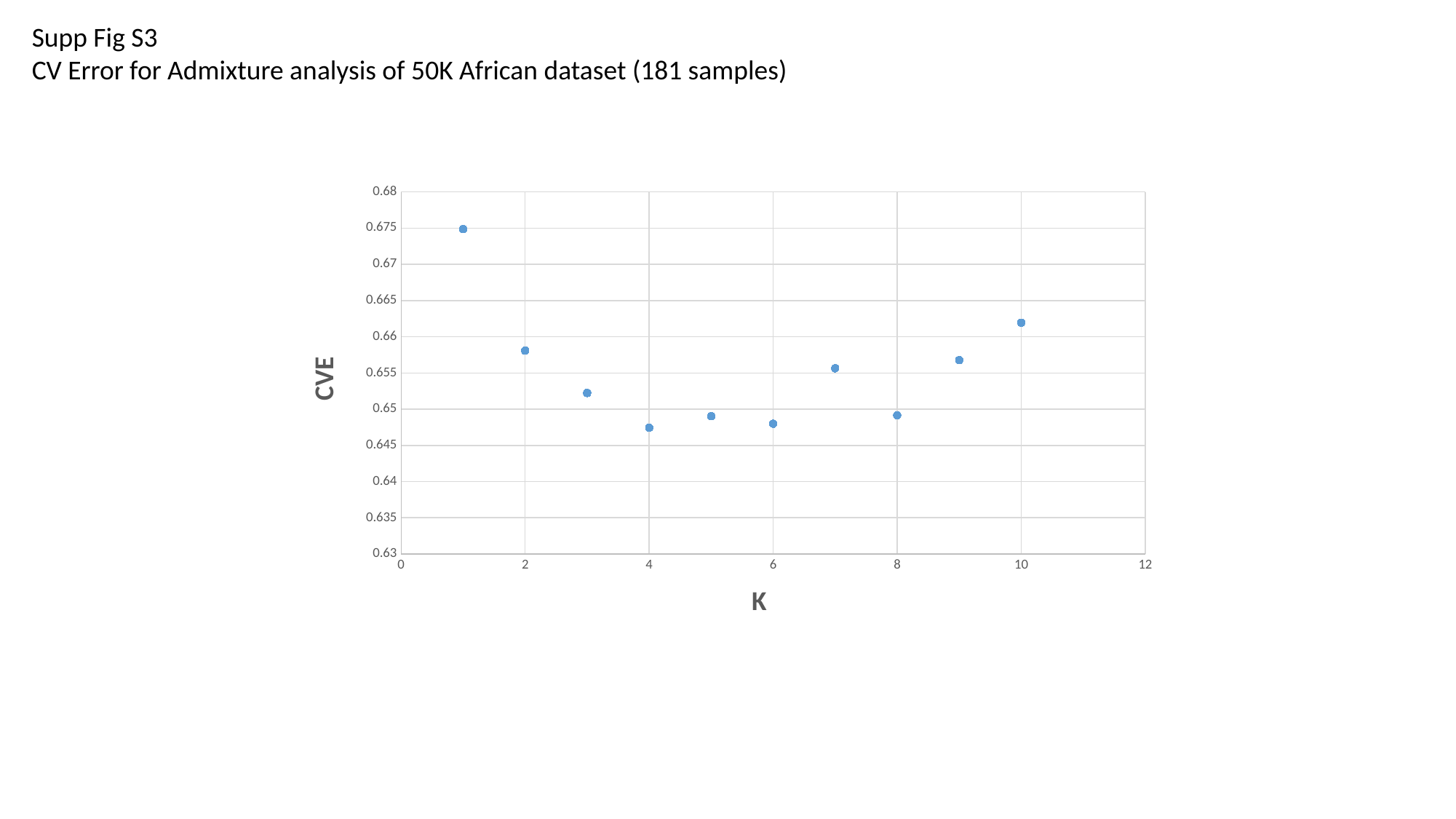

Supp Fig S3
CV Error for Admixture analysis of 50K African dataset (181 samples)
### Chart
| Category | |
|---|---|

## Slide 4
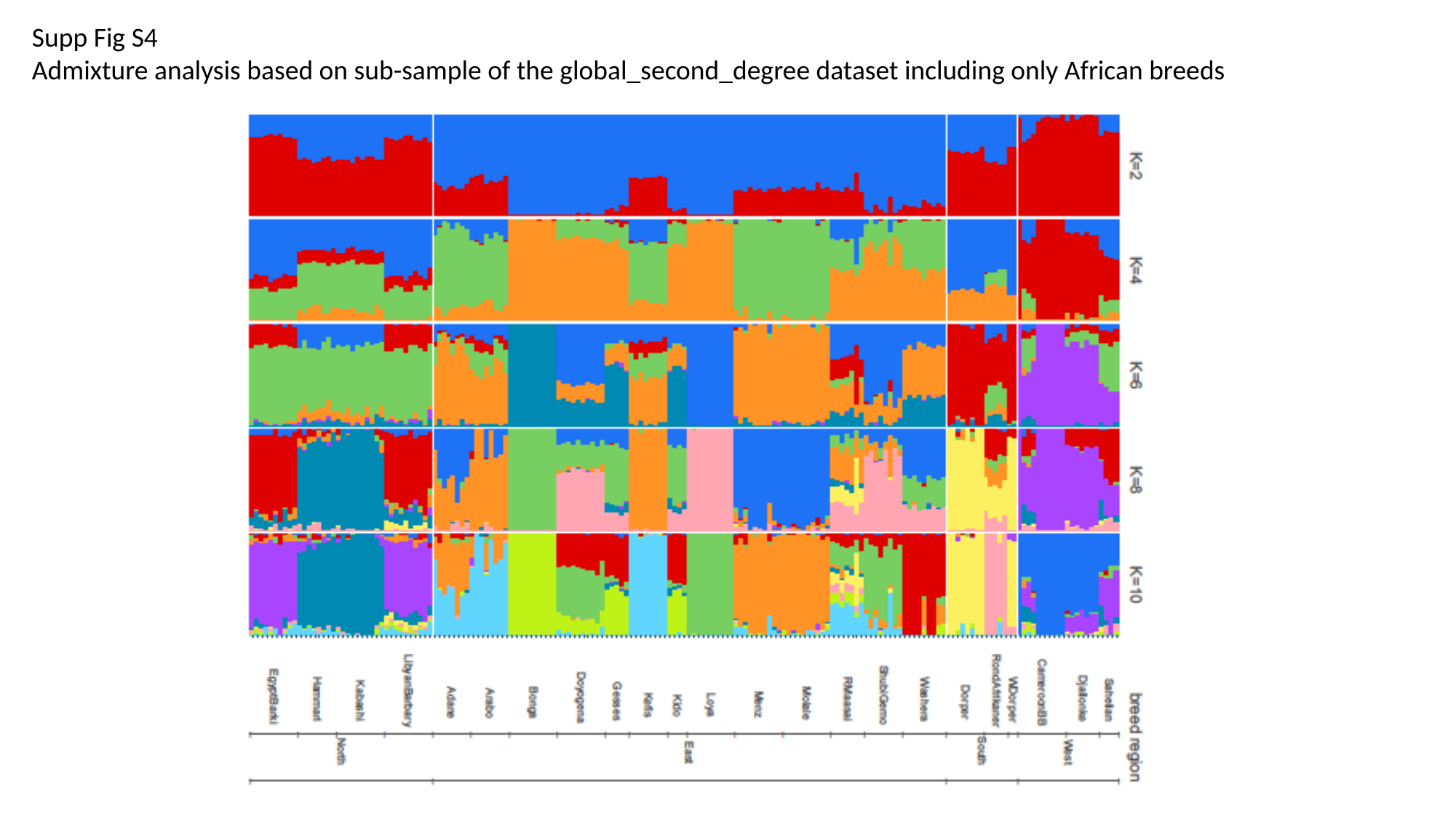

Supp Fig S4
Admixture analysis based on sub-sample of the global_second_degree dataset including only African breeds

## Slide 5
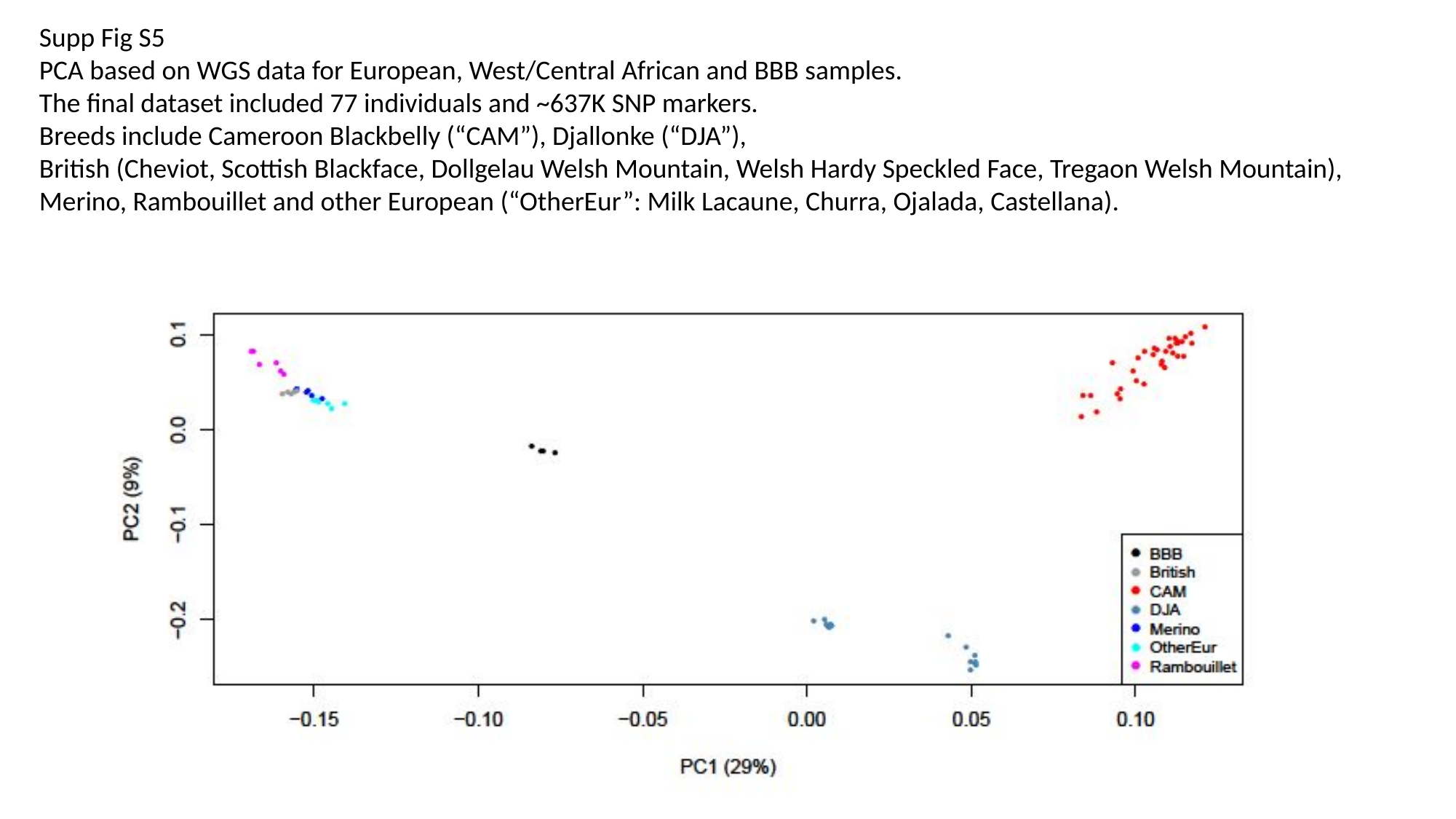

Supp Fig S5
PCA based on WGS data for European, West/Central African and BBB samples.
The final dataset included 77 individuals and ~637K SNP markers.
Breeds include Cameroon Blackbelly (“CAM”), Djallonke (“DJA”),
British (Cheviot, Scottish Blackface, Dollgelau Welsh Mountain, Welsh Hardy Speckled Face, Tregaon Welsh Mountain),
Merino, Rambouillet and other European (“OtherEur”: Milk Lacaune, Churra, Ojalada, Castellana).

## Slide 6
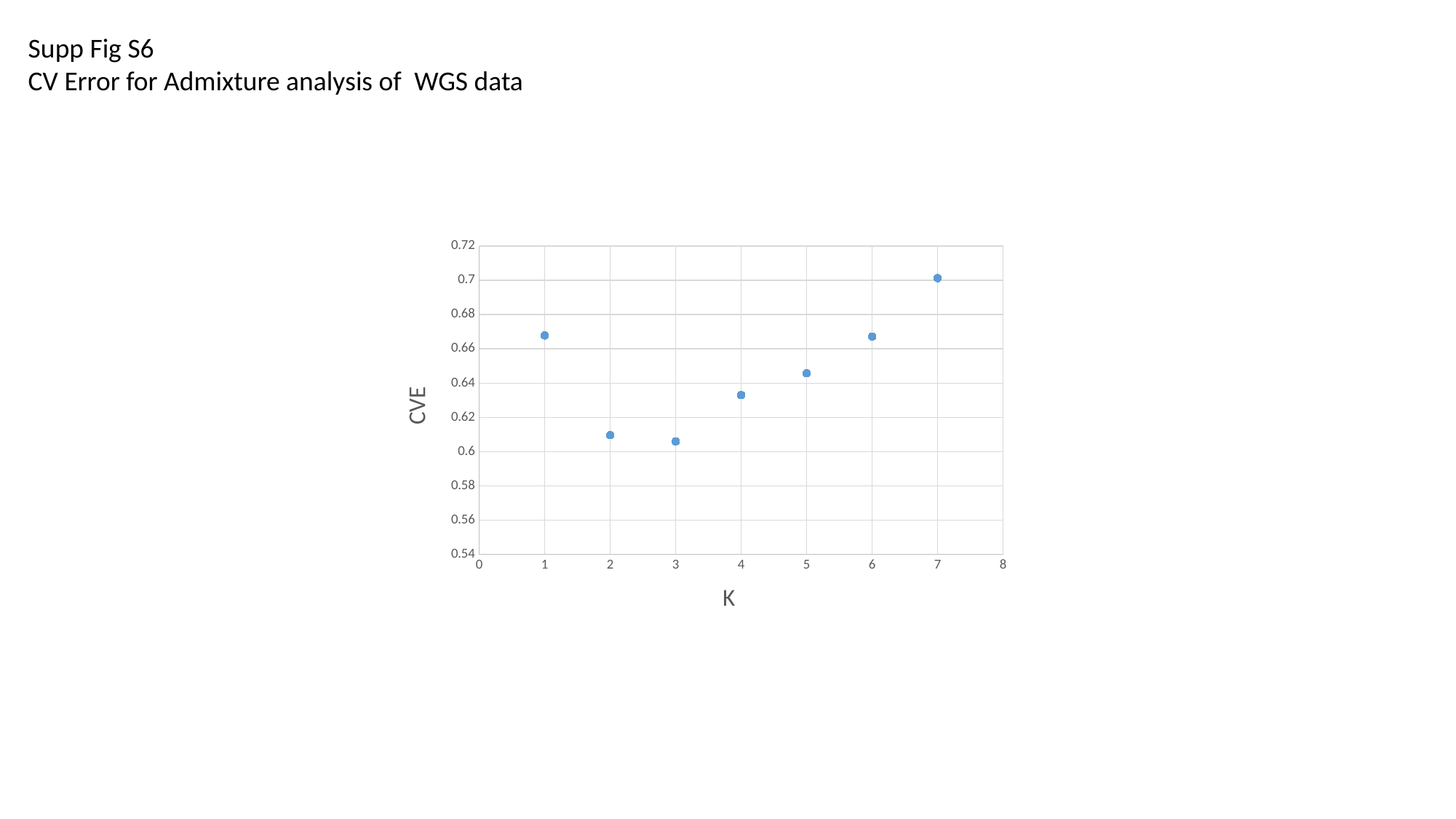

Supp Fig S6
CV Error for Admixture analysis of WGS data
### Chart
| Category | |
|---|---|

## Slide 7
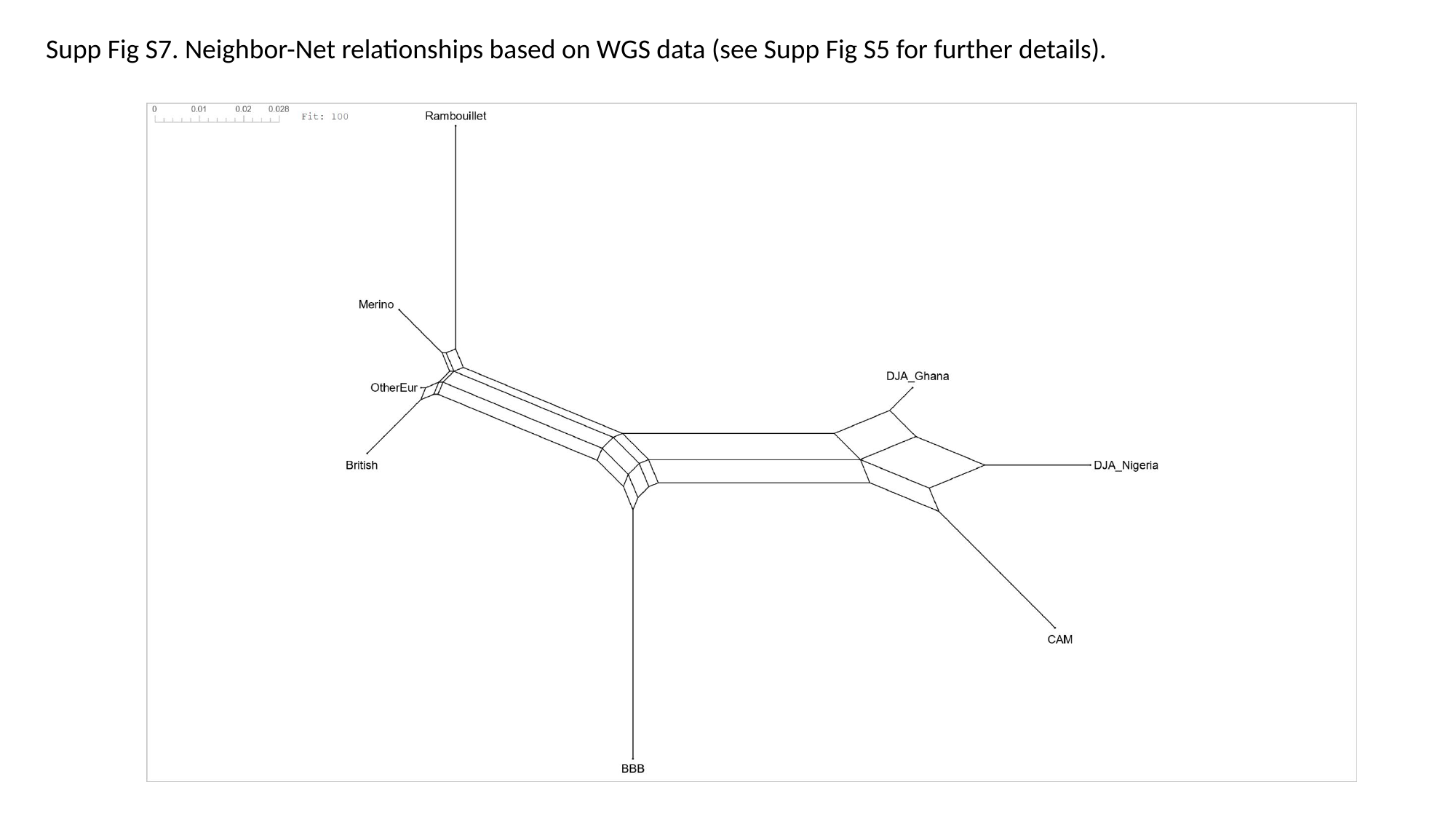

Supp Fig S7. Neighbor-Net relationships based on WGS data (see Supp Fig S5 for further details).

## Slide 8
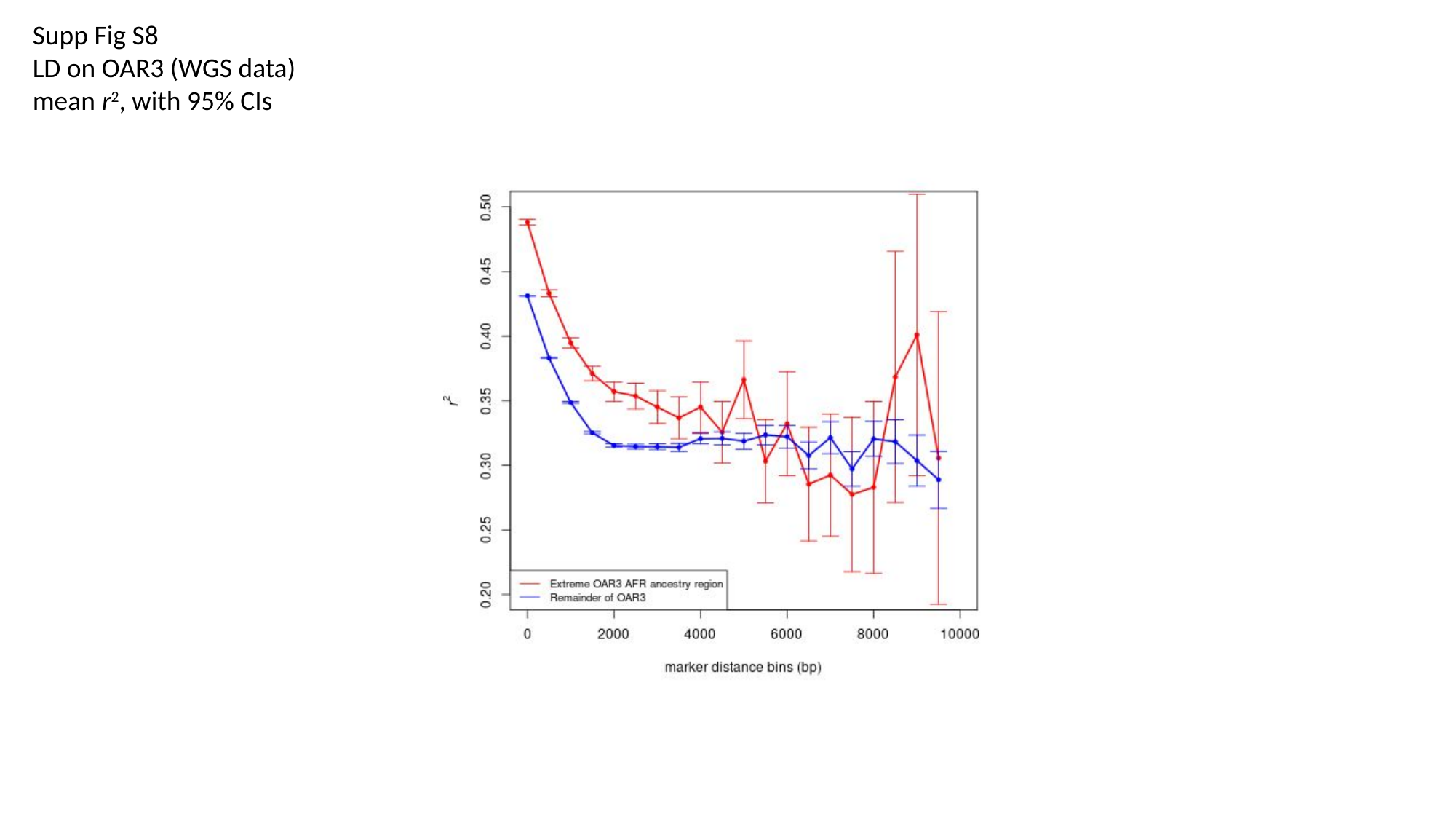

Supp Fig S8
LD on OAR3 (WGS data)
mean r2, with 95% CIs
